# Leveraging adenosine triphosphate for cancer theranostics

**DOI:** 10.7150/thno.106291

**Published:** 2025-03-24

**Authors:** Yuhan Li, Xinyu Zhang, Jianhua Wang, Kexin Wang, Boya Li, Xianghe Qiao, Wei He, Jinghua Cai, Danfeng Liu, Lei-Lei Yang

**Affiliations:** Department of Stomatology, The First Affiliated Hospital of Zhengzhou University, Zhengzhou 450052, China.

**Keywords:** Adenosine triphosphate, Tumor microenvironment, ATP-based therapy, ATP response, Cancer theranostics

## Abstract

Manipulation of the biochemical composition of the tumor microenvironment (TME) is a thriving research area in cancer treatment. Adenosine triphosphate (ATP), a key biochemical component, serves as an energy source for cancer cell proliferation. Notably, ATP can also act as a potent signal transducer to prime anti-tumor immune responses. There is increasing attention given to both the tumor-promoting and tumor-inhibiting roles of ATP in the context of possible new treatments for cancer. ATP levels in the TME are known to be significantly greater than in non-tumor tissues. This disparity presents an opportunity to exploit the ATP response for the delivery of anti-tumor drugs and tumor diagnosis. In this article, we provide a comprehensive overview of the existing strategies and mechanisms for ATP-based therapy and cancer diagnosis. We also discuss the current challenges in the field and propose potential areas for future research, to provide researchers with insights to further investigate the potential of ATP in cancer theranostics.

## 1. Introduction

In the tumor microenvironment (TME), adenosine triphosphate (ATP) serves as one of the major biochemical constituents, with a concentration as much as 10,000 times higher than in normal tissues 1, 2. ATP has been shown to play a pivotal role in the intricate and precise regulation of the TME 3. Incessant production of intracellular ATP (iATP) mainly acts as the energy currency in all biological processes of tumor that facilitates the survival, proliferation, and evasion of neoplastic cells 4. In addition to the well-established role as the energy carrier, ATP secreted or generated in the extracellular milieu can serve as a pivotal extracellular messenger 2. Extracellular ATP (eATP) elicits an anti-tumor immune response by interacting and activating multiple types of immune cells within the TME, thereby stimulating the anti-tumor immune response 5. However, eATP can be hydrolyzed to extracellular adenosine (eADO), which plays a contrary role in that it suppresses anti-tumor immunity through inhibiting the function of T cells, NK cells, and antigen-presenting cells 6. Normally, the balance of eATP and eADO maintains immune homeostasis in tissues 7. However, tumor cells tend to express high levels of extracellular ATP-hydrolyzing enzymes, which tips the balance towards the immune-suppressing function of eADO, thus fostering tumor progression 8. Based on the high concentration and fine-tuning of the regulatory role of ATP in the TME, developing strategies to leverage ATP for cancer therapy has long been a research focus and has recently become a hot topic.

## 2. The multifaced role of ATP in tumor microenvironment

**Figure 2:**
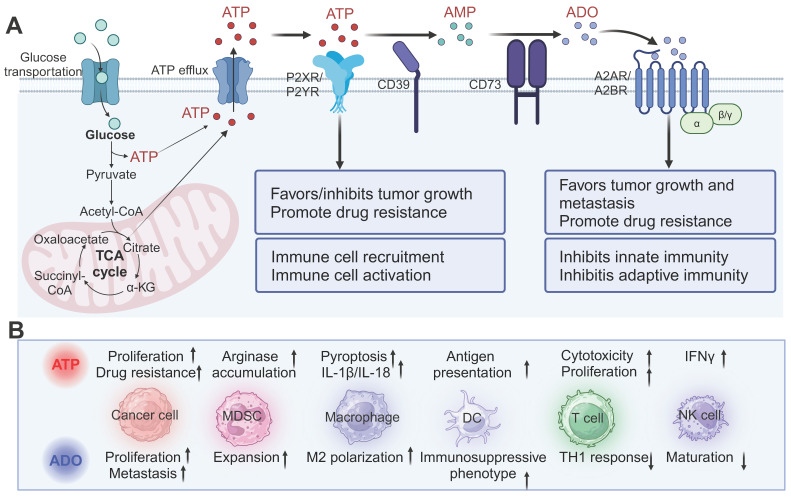
** The multifaceted role of ATP in the TME.** (A) ATP generated through glycolysis and the tricarboxylic acid cycle is released into the TME and can either stimulate inflammatory responses via P2X and P2Y purinergic receptors or be degraded into immunosuppressive ADO through the sequential actions of CD39 and CD73. ATP exerts both pro-tumor and anti-tumor effects, while ADO primarily promotes tumor progression, metastasis, and immune evasion by acting on A2A and A2B receptors. (B) The effects of ATP and ADO on various cell types in the TME. ATP enhances the proliferation of cancer cells, drug resistance, and immune cell activation, while ADO promotes tumor cell proliferation, metastasis, and immune suppression, influencing immune cell recruitment and polarization. MDSC: myeloid-derived suppressor cells; DC: dendritic cells; NK cells: natural killer cells; IFNγ: interferon-γ. Created with BioRender.com.

ATP, as the intracellular energy currency, is closely related to the growth and proliferation of tumor cells, playing a crucial role in cellular processes such as DNA replication, transcription, and protein synthesis 9. Additionally, ATP can be released into the extracellular space as a signaling molecule, significantly influencing the behavior of both tumor cells and immune cells (Figure 2) 10. Firstly, ATP can activate multiple survival signaling pathways in tumor cells by stimulating P2 receptors including P2Y1R, P2Y2R, P2Y6R, and P2X7R, which increase intracellular Ca^2+^ concentration and activate PI3K-AKT and ERK-MAPK pathways, thereby promoting cancer cell growth 11, 12, 13. Additionally, the immense energy demand of tumor cells during proliferation process could also be regulated by ATP 13, 14. Specifically, through its action on P2X7R, ATP regulates intracellular Ca^2+^ concentration and thereby upregulates glucose transportation by the transporter GLUT1 and at the meantime stimulates aerobic glycolysis by activating the related enzymes, thereby enhancing tumor cell metabolism 15-18. In addition to regulating tumor cells, eATP can also act as a “find-me” signal, recruiting neutrophils, macrophages, dendritic cells (DCs), and other immune cells into the TME 19, 20. Subsequently, eATP regulates immune cells through various mechanisms 21. DCs express several P2 receptors, particularly P2X7R, making them a finely tuned target for ATP regulation 22. When DCs are exposed to high concentrations of ATP, sufficient activation of P2X7R leads to the release of large amounts of IL-1β, which supports TH1-type immune responses and enhances antitumor immunity 23, 24. For macrophages, high concentrations of eATP can stimulate the production of cytokines such as IL-1α, IL-1β, IL-6, IL-18, CCL-2, and TNF-α through P2X7R activation 25-27. Additionally, higher concentrations of eATP can trigger pyroptosis in macrophages, leading to the release of pro-inflammatory substances that activate antitumor immunity 28. For T cells, ATP regulates migration, proliferation, and activation through calcium signaling mediated by both P2Y and P2X receptors 29, 30. Specifically, eATP acts as a potent chemoattractant, recruiting T cells via P2Y receptor-mediated signaling pathways 31. Moreover, P2X7R is ubiquitously expressed on the T cell surface, and activation of P2X7R by eATP not only induces T cell proliferation but also enhances their activation 32. Studies have shown that eATP can regulate the metabolism of memory CD8^+^ T cell populations, promoting their survival through P2X7R activation 33. Importantly, ATP has been shown to suppress Treg function by promoting FOXP3 degradation or directly reducing Treg numbers via P2X7R-mediated cytotoxic effects, thus alleviating immune suppression in the TME 34-36. Regarding NK cells, studies indicate that eATP-mediated activation of P2X7R and subsequent inflammasome formation are essential for NK cell-mediated metastasis control 37. Targeting CD39 to increase eATP has been shown to enhance IFN-γ production in NK cells and reduce tumor metastasis 37.

However, eATP is degraded into immunosuppressive adenosine (ADO) by ectonucleotidases, including ectonucleoside triphosphate diphosphohydrolase-1 (CD39), which hydrolyzes ATP to adenosine 5′-monophosphate (5′-AMP), and ecto-5′-nucleotidase (CD73), which further converts 5′-AMP into ADO 8, 38, 39. CD39 and CD73 are highly expressed in various cell types within the TME, including tumor cells, stromal cells, endothelial cells, infiltrating immune cells, and others 40, 41. The generated ADO can bind to ADO receptors A2AR and A2BR on the surface of various immune cells. Activation of these receptors suppresses immune cell activity, promoting macrophage polarization to the M2 phenotype, inhibiting the antigen-presenting function of DCs, suppressing TH1 responses, and impairing NK cell maturation. This cascade of events contributes to immune suppression and tumor progression 30, 41-43.

In summary, ATP plays a multifaced role in the TME. It not only serves as an intracellular energy source to support the growth, proliferation, and metabolism of tumor cells but also triggers a series of survival and metabolic signaling pathways through the activation of P2 receptors on tumor cells. Meanwhile, eATP acts as a signaling molecule to attract and activate immune cells, thereby enhancing antitumor immune responses. However, once ATP is degraded by CD39 and CD73 into ADO, it binds to A2A/A2B receptors to inhibit immune cell functions and promote the expansion of regulatory T cells, leading to immune suppression and tumor progression. This series of complex mechanisms underscores the critical role of ATP in tumor development.

## 3. Depletion of iATP to starve tumors

**Figure 3:**
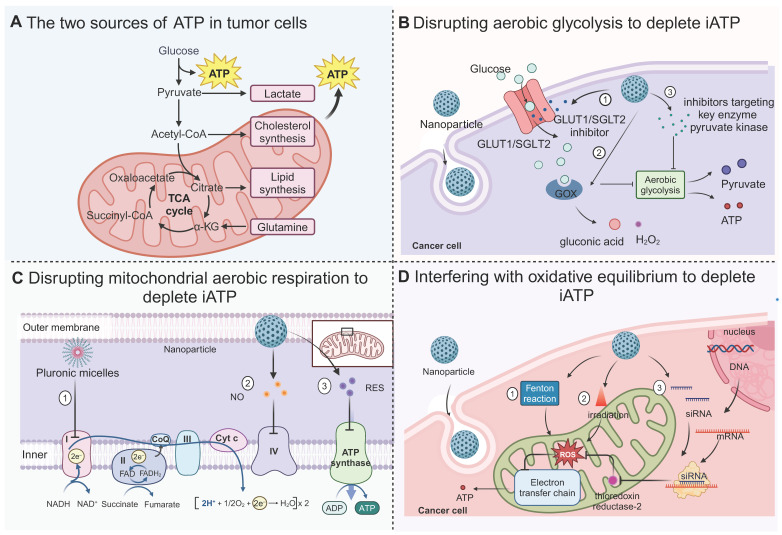
** Strategies for depleting intracellular (iATP) to starve cancer cells.** (A) Glycolysis and oxidative phosphorylation are the main processes that generate ATP for tumor cells. (B) Limiting glucose utilization to disturb aerobic glycolysis through (1) introduction of nanoparticles carrying GLUT1/SGLT2 inhibitors, which block import of glucose; (2) glucose oxidase, which depletes the supply of glucose and inhibit aerobic glycolysis; (3) inhibitors of key enzyme pyruvate kinase. (C) Several strategies to interfere with mitochondrial oxidative phosphorylation. Micellar polymers inhibit the activity of the electron transport chain. The NO released by nanoparticles inhibits complex IV and resveratrol (RES) inhibits complex V. (D) Different methods to maintain a high level of reactive oxygen species (ROS) for disrupting oxidative balance. ROS can be increased through Fenton reaction, irradiation, activation of superoxide dismutase or inhibition of toxin. Created with BioRender.com.

In contrast to healthy cells, tumor cells predominantly generate iATP through the Warburg effect 44. However, the existing literature suggests that oxidative phosphorylation is also an important pathway for iATP production (Figure 3A) 45. Because iATP serves as a primary energy reservoir for cellular proliferation, invasion, and metastasis 46, it exhibits a complex interplay with the fundamental energy provision in cancer cells, the maintenance of multidrug resistance (MDR) within cancer cells, and the synthesis of heat shock proteins (HSPs) that help cancer cells maintain low sensitivity to photothermal therapy (PTT). Depletion of iATP can exert inhibitory effects on cancer cell growth and differentiation, while enhancing the susceptibility of tumor cells to chemotherapy and PTT. Currently, the research focus in this area is on blocking the source of ATP (Figure 3B-D).

### 3.1 Disrupting aerobic glycolysis to deplete iATP

Even in the presence of oxygen, tumor cells exhibit enhanced glucose consumption through aerobic glycolysis, thereby generating sufficient ATP to sustain their metabolic demands. This phenomenon is referred to as the Warburg effect 47, 48. However, glucose is not efficiently utilized during glycolysis, necessitating a substantial quantity of glucose substrate to generate an adequate ATP yield in tumor cells 49. Consequently, the high demand of energy necessitates the high expression of glucose transporters (GLUTs) in the tumor cell membrane 50. The utilization of glucose oxidase (GOX) or glucose transporter inhibitors can effectively mitigate ATP production by lowering substrate availability for aerobic glycolysis 51, 52. Targeting glycolytic enzymes with inhibitors can also effectively disrupt the energy supply through the glycolytic pathway in cancer cells, resulting in growth arrest, suppression of proliferation, and initiation of programmed apoptosis 53. With these findings in mind, we conducted a comprehensive survey of recent research to elucidate the impact of inhibiting intratumor glycolysis on ATP depletion by targeting GLUTs and glycolytic enzymes and utilizing GOX.

#### 3.1.1 Interfering with glucose transporters

Given that glucose is the main source of energy for tumor cells 54, it is imperative that tumor cells express GLUTs on their plasma membranes. The translocation of glucose across the plasma membrane, which represents a crucial rate-limiting step in its metabolic utilization, is facilitated by GLUTs 55. GLUTs consist of 12 transmembrane transporters. A total of six members have been reported thus far: GLUT1-5 and GLUT7 56. GLUT1 expression is frequently upregulated in a variety of solid tumors and hematologic malignancies and exhibits superior glucose affinity compared to other GLUT transporters 57. Consequently, GLUT1 plays a pivotal role in tissues that rely heavily on glucose as their primary energy source 58, 59. Diclofenac has been shown to deplete GULT1 level on the surface of tumor cell membranes, thereby inhibiting glucose uptake, blocking glycolysis, reducing ATP levels, and impeding the expression of HSPs in tumor cells, thus enhancing the sensitivity of PTT 60. Researchers have developed CD44-targeted functional polymers for the decoration of plasma-gold nanorods. By specifically targeting CD44, sequential time-dependent release of Diclofenac can be achieved through the triggering of hyaluronidase, which is abundantly present in tumor tissues 61. As a result, ATP levels in tumor cells are reduced.

#### 3.1.2 Inhibiting aerobic glycolytic enzyme activity

Pyruvate kinase, a pivotal enzyme in aerobic glycolysis, facilitates the conversion of phosphoenolpyruvate and ADP to pyruvate and ATP, respectively, thus exerting regulatory control over tumor cell metabolism 62. Repression of ATP production through the use of inhibitors targeting pyruvate kinase has been demonstrated to induce tumor cell necrosis or apoptosis 53. A recent study has demonstrated that a folate-modified silver nanoparticle carrying albendazole effectively inhibits ATP production in tumor cells 63. The inhibitory effect of albendazole on the glycolytic pathway is achieved by attenuating the enzymatic activity of pyruvate kinase, which inhibits cancer cells 64. Inhibition of the glycolytic pathway alone leads to a compensatory increase in mitochondrial metabolism 65; therefore, synergistic therapy with glycolysis inhibitors and mitochondrial inhibitors is more effective 63, 65. Thus, silver nanoparticles were introduced in a study, where silver nanoparticles effectively attacked and disrupted the mitochondria and synergized with albendazole, further inhibiting ATP production 63. Additionally, a significant amount of lactic acid is generated within the glycolytic pathway, where it acts as a pro-tumor factor 66. Thus, the combination of lactate oxidase and inhibitors targeting glycolytic enzymes can effectively reduce lactate production and eliminate residual lactic acid; this combination effect blocks ATP production and enhances anti-tumor efficacy 67.

In addition to pyruvate kinase inhibitors, compounds targeting other key enzymes in glycolysis also demonstrate promising therapeutic potential. For instance, 12-O-deacetyl-phomoxanthone A (12-ODPXA), a xanthone compound, inhibits ovarian cancer growth and metastasis by targeting pyruvate dehydrogenase kinase 4 (PDK4) 68. By suppressing glycolysis and epithelial-mesenchymal transition (EMT), 12-ODPXA effectively reduces tumor cell proliferation and migration. Zebrafish xenograft models confirmed the efficacy, highlighting 12-ODPXA as a promising candidate for metabolic therapy in ovarian cancer.

#### 3.1.3 Reducing the substrate of aerobic glycolysis

GOX catalyzes the oxidation of glucose, leading to the production of cytotoxic H_2_O_2_ and gluconic acid in tumor cells 69, 70. This process effectively depletes glucose in tumor cells, thereby reducing substrate availability for aerobic glycolysis and inhibiting ATP generation 70. Consequently, tumor growth is stunted by obstructing the supply of nutrition and energy 71. A double-enzyme-catalyzed nanosystem containing Pt nanoparticles and GOX was developed to reduce ATP production by catalyzing glucose 51. Within the nanosystem, GOX degrades glucose into H_2_O_2_ and Pt catalyzes H_2_O_2_ to produce oxygen, which in turn participates in and facilitates the process of GOx-mediated glucose consumption 51. The consumption of glucose by these nanosystems results in a severe shortage of ATP, creating an energy-deficient microenvironment that induces intracellular damage during the GOX-mediated starvation process, ultimately facilitating the eradication of cancer cells 51. The synergistic combination of GOX and Pt were also conducted in multiple studies for starvation therapy 72-75. In addition to reducing glucose for ATP depletion, researchers have employed lipase inhibitors to disrupt ATP turnover, consequently increasing the cytotoxicity of tumor cells and reducing the viability of cancer cells 76.

In PTT, photothermal agents are applied to convert light energy into thermal energy, thereby facilitating tumor tissue eradication 77, 78. However, during PTT, tumor cells can upregulate the expression of HSPs, which, in conjunction with ATP, impairs PTT 78. Thus, the inhibition of ATP production by GOX in tumors helps overcome HSP-dependent tumor resistance, thereby enhancing the efficacy of PTT 78. As an example, Hu *et al.* developed a light-responsive liquid metal nanoparticle enzyme system that synergistically integrates cancer starvation therapy with PTT for enhanced tumor treatment 69. Leveraging the photothermal conversion properties of liquid metals, the system enabled precise modulation of GOX catalytic efficiency under near-infrared (NIR) light irradiation 69. *In vitro* studies revealed that ATP levels in tumor cells were significantly reduced by 14% and 67% after 12 and 24 hours of incubation with GOX, respectively, compared to untreated controls. This substantial ATP depletion disrupted essential cellular processes, including energy production required for proliferation and the synthesis of HSPs, which are crucial for maintaining tumor cell survival under thermal stress. Importantly, subsequent NIR irradiation further exacerbated ATP depletion, intensifying HSP suppression and increasing tumor cell sensitivity to PTT-induced cytotoxicity. *In vivo* experiments provided robust evidence that this nano system effectively eradicated cancer cells and inhibited solid tumor growth under NIR irradiation. The combined treatment demonstrated significantly superior anti-tumor efficacy compared to monotherapies involving either tumor starvation or PTT alone. This enhanced therapeutic outcome was attributed to the synergistic effects of ATP depletion and oxidative stress induced by GOX-catalyzed glucose oxidation. Specifically, ATP depletion impaired the tumor cells' ability to counteract thermal damage by downregulating HSP expression. These results underscore ATP depletion as a critical mediator in the integration and optimization of PTT and starvation therapy, demonstrating its pivotal role in overcoming tumor resistance mechanisms. This study highlights a promising framework for improving therapeutic outcomes in challenging and treatment-resistant cancers by leveraging ATP depletion 69.

In the development of strategies to disrupt aerobic glycolysis and deplete iATP in tumor cells, nanomaterial-based designs have played a pivotal role by leveraging their unique properties to enhance therapeutic efficacy and overcome tumor resistance. Key nanodesign strategies include functionalized nanocarriers, such as CD44-targeted polymers, enabling controlled drug release 61; synergistic nanocomposites, such as dual-enzyme nanosystems combining GOX and platinum nanoparticles, which facilitate substrate depletion and reactive oxygen species (ROS) generation 51; and light-responsive nanoparticles, such as liquid metal nanoparticles and rhenium-based nanomaterials, integrating starvation therapy with PTT for precise spatiotemporal control 52, 69. The materials employed in these designs are diverse and include metallic nanoparticles (e.g., gold, silver, and platinum) for enhancing enzyme stability and specificity, carbon-based nanomaterials (e.g., graphene oxide and carbon dots) for high drug-loading capacity and photothermal conversion, polymeric nanoparticles for selective and sustained drug delivery, and hybrid nanocomposites (e.g., Re@ReP-G) that combine enzymatic degradation and photothermal activation to amplify anti-tumor effects 51, 52, 69, 79. These nanotechnological innovations have significantly advanced ATP-depletion-based therapeutic approaches, providing promising avenues for enhancing biocompatibility, minimizing off-target effects, and improving clinical translational potential.

### 3.2 Disrupting mitochondrial aerobic respiration to deplete iATP

Mitochondrial respiration encompasses aerobic glycolysis and mitochondrial aerobic respiration 80. The results of numerous studies have underscored the reliance of tumor cells on the energy derived from aerobic glycolysis 81. Nevertheless, there is also evidence that mitochondrial aerobic respiration is a principal source of metabolic energy 82. The electrons liberated during oxidative phosphorylation, which are associated with mitochondrial aerobic respiration, are transferred to oxygen (O_2_) through proton pumps in the respiratory chain 83. These proton pumps, namely complex I to complex IV, establish an H^+^ gradient within the inner mitochondrial membrane, where complex IV plays a critical role in facilitating electron transport along the chain and complex V directly catalyzes ATP synthesis 83, 84. Disruption of the respiratory chain can result in mitochondrial dysfunction and impede ATP synthesis within mitochondria, consequently affecting DNA replication, cell proliferation, and drug resistance of tumor cells 85, 86.

#### 3.2.1 Inhibiting complex IV

During mitochondrial oxidative phosphorylation, complex IV functions as a proton pump and plays a pivotal role in the electron transport chain 87, 88. The consumption of O_2_ by the mitochondrial complex IV constitutes 90% of the total O_2_ utilization, which is indispensable for ATP synthesis. When complex IV is inhibited, the blockage of electron transfer leads to mitochondrial dysfunction and subsequent ATP depletion 87. Studies have demonstrated that high concentrations of NO exert an inhibitory effect on cytochrome c oxidase, a component of mitochondrial complex IV 89, 90. Researchers have developed a sodium polyglycolide nanoparticle with an outer layer containing the photosensitizer chlorin e6 and L-arginine (L-Arg) as the donor of nitric oxide (NO). Upon laser irradiation, chlorin e6 induces ROS formation, which oxidize L-Arg to NO and inhibit compound IV 80. Inhibition of mitochondrial respiration mediated by NO reduces multi-active oxygen metabolism in tumor cells, enhances the hypoxic state of the TME, depletes ATP in tumor cells, and inhibits the proliferation of tumor cells 80.

#### 3.2.2 Inhibiting complex V

Complex V directly catalyzes ATP synthesis; inhibiting complex V has been demonstrated to be more effective than inhibiting complex IV 83. Resveratrol (RES) is an inhibitor of ATP synthetase that can interfere with the metabolism of the respiratory chain of tumor cells and trigger the blockage of ATP production 91. Researchers have utilized nano-methods to more effectively deliver RES into mitochondria and achieve an inhibitory effect of RES on the respiratory chain. RES has been incorporated into a porous coordination polymer (PCN-224) 92. PCN delivery endows nanoparticles with the ability to target mitochondria. The ATP-coordinated decomposition of the polychlorinated naphthalene structure leads to the release of the trapped drug RES, which accumulates in the mitochondria and inhibits complex V-mediated ATP synthesis for metabolic therapy 92.

#### 3.2.3 Reducing the activity of the electron transfer chain

Pluronic acid acts as a nonionic polymeric detergent that reduces oxidative metabolism in tissues, cells, and isolated mitochondria. In 1992, researchers first observed that Jurkat T lymphoma cells were depleted of ATP in lymphoma cells 2 h after exposure to the Pluronic block copolymer P85 93. In 2000, researchers observed that P85 and P105 depleted iATP and reduced the activity of electron transport chains in the mitochondria 94. Further research revealed that Pluronic ATP depletion could be caused by a variety of factors, including their ability to act as K^+^ ionophores, uncoupled oxidative phosphorylation, or the direct inhibition of nicotinamide adenine dinucleotide (NADH) dehydrogenase complexes by interacting with hydrophobic sites located in complexes in mitochondrial membranes 95.

P-glycoprotein (P-gp) is a well-known transporter protein belonging to the ATP-binding cassette (ABC) family of membrane transporters, which actively expels drugs from tumor cells in an ATP-dependent manner and consequently mediates drug resistance 96. Experimental findings from a study on drug-loaded lipid-based nanoparticles showed that pluronic-induced rapid ATP depletion could effectively inhibit the P-gp efflux system by disrupting its energy supply. This inhibition does not cause cytotoxicity in either multidrug-resistant or -sensitive cells. However, when combined with chemotherapeutic agents, ATP depletion significantly enhances their cytotoxic effects through specific mechanisms. ATP depletion reduces P-gp activity, leading to increased intracellular retention of chemotherapeutic drugs. Furthermore, ATP depletion imposes energy stress on tumor cells, as evidenced by changes in mitochondrial membrane potential and structure, which compromises their ability to maintain metabolic functions and repair damage caused by chemotherapeutic agents. Notably, P-gp-overexpressing tumor cells are more significantly affected by ATP depletion compared to non-resistant cells, as they require higher energy levels to sustain both drug efflux and their elevated metabolic demands. These findings demonstrate that nanoparticle-induced ATP depletion synergizes with chemotherapy to overcome drug resistance and improve therapeutic efficacy 96.

Strategies targeting mitochondrial aerobic respiration to deplete iATP in tumor cells have employed innovative nanomaterial-based approaches, effectively disrupting the electron transport chain and ATP synthesis. One prominent strategy involves the inhibition of mitochondrial complex IV, which is responsible for 90% of oxygen consumption required for ATP synthesis. Sodium polyglycolide nanoparticles functionalized with chlorin e6 and L-Arg generate ROS and NO upon laser activation, thereby blocking complex IV activity, inducing mitochondrial dysfunction, and exacerbating tumor hypoxia 97. Similarly, complex V-mediated ATP synthesis has been targeted using PCN-224 loaded with RES 92. The ATP-coordinated decomposition of PCN ensures efficient mitochondrial targeting and the controlled release of RES, ultimately inhibiting ATP production for metabolic therapy. Additionally, Pluronic block copolymers (e.g., P85 and P105) disrupt ETC activity through mechanisms such as uncoupling oxidative phosphorylation, acting as K+ ionophores, and inhibiting NADH dehydrogenase complexes, resulting in ATP depletion 95. This ATP depletion suppresses the ATP-dependent P-gp efflux system, significantly enhancing the efficacy of chemotherapeutic agents in drug-resistant tumor cells without inducing significant cytotoxicity. The nanomaterials utilized, including polymer-based nanoparticles, photosensitizers, small-molecule inhibitors, and block copolymers, enable precise targeting of mitochondrial functions and synergistic therapeutic effects, offering significant potential for advancing mitochondrial-targeted metabolic therapies and overcoming drug resistance in cancer treatment.

### 3.3 Interfering with oxidative equilibrium to deplete iATP

ROS, which are generated as byproducts of normal cellular metabolism, play an essential role in maintaining homeostasis and mediating signaling pathways 98. Compared with normal cells, tumor cells exhibit significantly elevated ROS levels 98. ROS production activates pro-tumor signaling and improves cell survival and proliferation; however, higher concentrations of ROS can induce oxidative stress, leading to cell damage 98. Tumor cells detoxify by expressing high levels of antioxidant proteins, such as antioxidant enzymes, superoxide dismutase (SOD), catalase, glutathione peroxidase, and glutathione transferase, while maintaining pro-tumor signaling and resistance to apoptosis 99, 100. Mitochondria play a crucial role in endogenous ROS production within tumor cells 101, 102. However, mitochondria are susceptible to damage due to increased ROS generation 84. Thus, ROS can attenuate ATP production by inducing oxidative stress and compromising mitochondrial functionality. Additionally, ROS exert antitumor effects through energy blockade or by reversing ATP-dependent MDR in cancer cells 103.

#### 3.3.1 Depleting ATP production by increasing ROS

**Figure 4:**
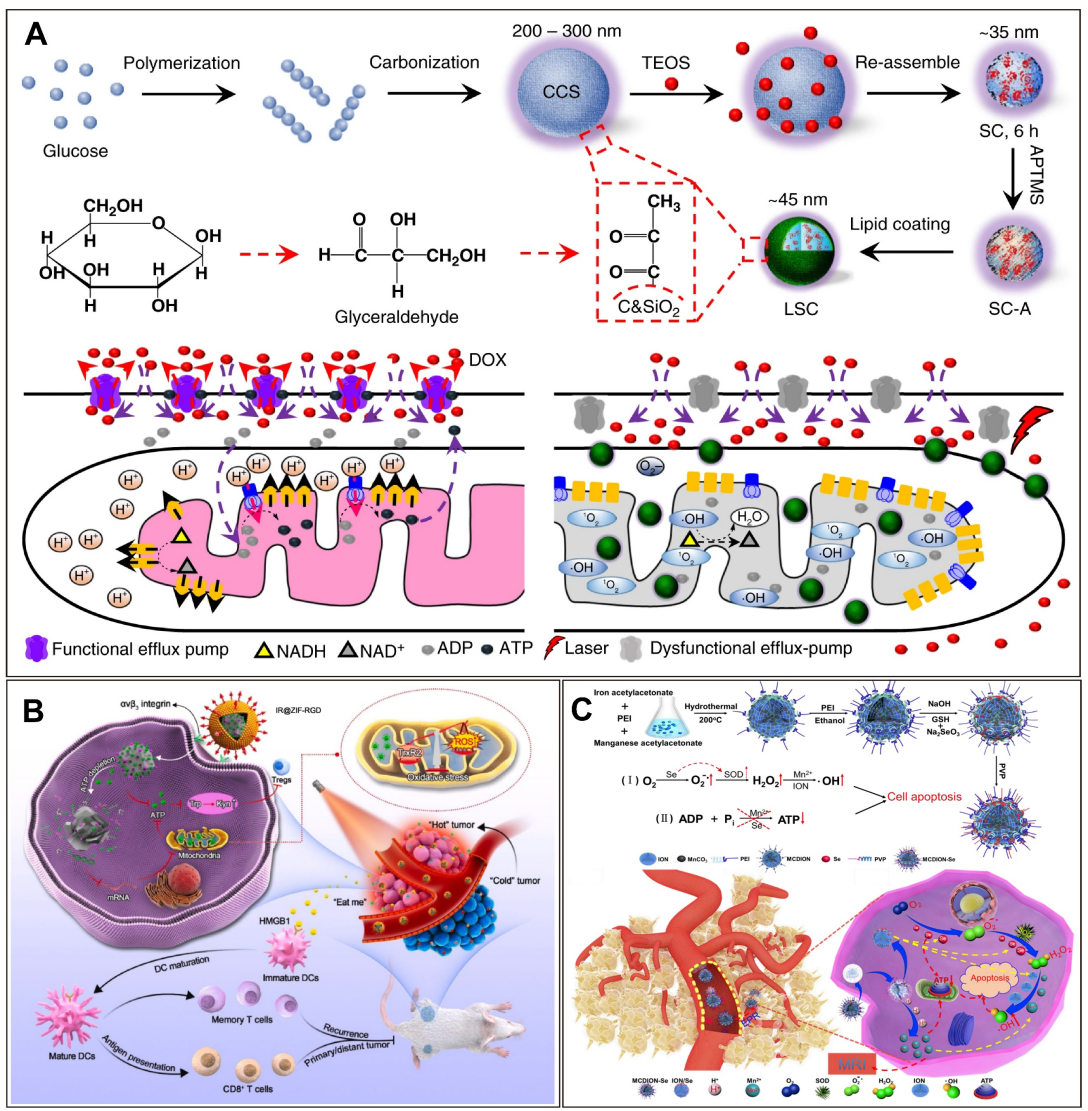
** Nanotechnology-enhanced cancer starvation therapy through depleting iATP.** (A) A lipid membrane-coated silica-carbon hybrid nanoparticle targets mitochondria and, under NIR laser irradiation, produce ROS that oxidize the NADH into NAD^+^. Adapted with permission from 105, copyright 2018 Springer Nature. (B) An ATP-exhausted nanocomplex (IR@ZIF-RGD); the component of ZIF-90 hydrolyzes and deplete iATP. Adapted with permission from 111, copyright 2022 Elsevier. (C) Nanosystem containing nano selenium and Mn^2+^ serves as ATP inhibitor and blocks the energy required for cancer cell growth. Adapted with permission from 110, copyright 2019 Elsevier. (Details of the mechanisms of each of the nanotechnologies can be found in the main text.).

Photodynamic therapy (PDT) has been used to generate ROS that inhibit iATP 104. For example, a type of lipid membrane-coated silica-carbon hybrid nanoparticles was developed, which selectively target mitochondria and generate ROS under NIR laser irradiation 105. This precise ROS generation directly oxidizes NADH to NAD+, leading to a depletion of ATP through disruption of the proton gradient essential for ATP synthase activity. Consequently, the ATP-dependent efflux pumps become dysfunctional, preventing drug efflux and effectively overcoming MDR in cancer cells. Notably, this effect persists for up to 5 days, providing a therapeutic window for enhanced chemotherapy efficacy 105. Moreover, ATP depletion significantly impacts the localization and activity of P-gp efflux pumps, which are primarily located on the plasma membrane under normal conditions. ATP not only fuels drug efflux but also plays a crucial role in the active transport of P-gp to the membrane. Upon ATP depletion, P-gp becomes redistributed into the cytoplasm, likely due to insufficient energy for its membrane localization (Figure 4A). This altered distribution reduces the number of functional P-gp pumps on the membrane, thereby enhancing intracellular drug retention. Furthermore, the combined treatment of nanoparticles and NIR laser irradiation effectively sensitized multidrug-resistant tumor cells to various chemotherapy agents, including doxorubicin, paclitaxel, and irinotecan. This indicates the potential for broad therapeutic application. These findings underscore the synergistic potential of ROS-induced ATP depletion with traditional chemotherapy by simultaneously targeting drug efflux mechanisms and disrupting intracellular energy metabolism.

In addition to PDT, the Fenton reaction also generates ROS 106. The Fenton reaction involves the reaction of Fe^2+^ with H_2_O_2_, resulting in the generation of hydroxyl radicals 106, 107. Hydroxyl radicals are known to induce oxidative stress in cells and reduce ATP production in mitochondria. Researchers have utilized metal polyphenol-coated calcium carbonate as a pH-responsive nanocarrier to facilitate tumor penetration and effectively reverse MDR 108. Fe²⁺ ions catalyze the Fenton reaction, significantly amplifying oxidative stress, which in turn disrupts mitochondrial-mediated ATP production. This reduction in ATP levels decreases the functionality of ATP-dependent drug efflux pumps, such as P-gp, thereby overcoming MDR 108. ATP depletion further impairs mitochondrial function and cellular metabolic processes, ultimately reducing tumor cell proliferation and survival. Furthermore, the inhibition of ATP-driven drug efflux enhances intracellular retention of chemotherapeutic agents, such as doxorubicin (DOX), within MDR cancer cells 108. *In vivo* studies have demonstrated that this nanosystem exerts a potent therapeutic effect by synergizing oxidative stress and chemotherapeutic mechanisms, leading to the effective suppression of DOX-resistant 4T1 tumor growth 108.

In chemodynamic therapy (CDT), hydroxyl radicals generated by Fenton-like reactions cause DNA and protein damage, thereby accelerating cell apoptosis 109. However, the low content of Fe^2+^ in cancer cells reduces the efficiency of the Fenton reaction, rendering the generated hydroxyl radicals insufficient to induce apoptosis in cancer cells 110. To solve this problem, researchers have used nanoselenium to simultaneously activate SOD and promote the production of superoxide anion radicals. Meanwhile, SOD catalyzes SOAR to H_2_O_2_, which provides sufficient substrates for Fenton-like reactions, thus boosting the efficiency of CDT. Nano-selenium inhibited ATP production, thereby blocking the energy supply to cancer cells 110. Experiments at the cellular level have shown that the ATP content of cells incubated with the nanosystem decreased significantly, accompanied by a certain degree of apoptosis (Figure 4C).

#### 3.3.2 Depleting ATP by inhibiting antioxidant enzymes

Thioredoxin reductase-2 (TrxR2) is a key mitochondrial antioxidant enzyme and its inhibition can lead to mitochondrial oxidative stress and tumor cell dysfunction 111. Oxidative stress amplified in tumor cells has been shown to consume iATP and inhibit ATP production 112. The ATP-depleted nanocomposite was composed of ATP-responsive zeolitic imidazole frameworks-90 (ZIF-90) and siRNA targeting TrxR2. TrxR2 reduces H_2_O_2_ to H_2_O and maintains ROS levels at physiological levels 112. siRNAs targeting TrxR2 can bind to the mRNA of TrxR2 and inhibit the expression of this reductase, thus increasing ROS production and amplifying oxidative stress. Thus, it can deplete ATP, inhibit ATP synthesis, and induce tumor metabolic disorder 111. In addition, ZIF-90 self-assembled from imidazole-2-carboxaldehyde (2-ICA), and Zn^2+^ reacted to degrade ATP, leading to ATP hydrolysis and depletion. Excess exogenous Zn^2+^ exhibits ATPase-like activity that induces ATP hydrolysis, which can further induce ATP depletion 111. The authors reported that ATP depletion impeded the Trp metabolism program, thereby facilitating the invasive potential of effector T cells in tumor progression 111. Amplified oxidative stress can be synergistically combined with photosensitizer-mediated PDT to induce the death of potent immunogenic cells, thereby augmenting anti-tumor immunogenicity. The findings from an animal experiment showed that the immunosuppressive status of the TME was reversed when the percentage of DCs, maturation rate, and activation of cytotoxic T lymphocytes increased (Figure 4B) 111.

Strategies targeting oxidative equilibrium to deplete iATP in tumor cells utilize advanced nanomaterial-based approaches to amplify oxidative stress, disrupt mitochondrial function, and inhibit antioxidant defenses. Tumor cells, characterized by elevated ROS levels, are particularly vulnerable to oxidative damage, which can compromise ATP production. PDT and Fenton reactions are prominent methods for ROS generation. Lipid membrane-coated silica-carbon hybrid nanoparticles and metal polyphenol-coated calcium carbonate carriers effectively disrupt mitochondrial ATP synthesis and reverse MDR 105, 108. Nanoselenium further enhances CDT by catalyzing the production of superoxide anion radicals and hydroxyl radicals 110. Additionally, targeting antioxidant defenses, such as TrxR2, using ATP-responsive zeolitic imidazole frameworks (ZIF-90) loaded with siRNA, amplifies oxidative stress, hydrolyzes ATP, and induces metabolic dysfunction 111. Beyond depleting ATP, these nanomaterials enhance immunogenic cell death (ICD) and boost anti-tumor immune responses by increasing dendritic cell maturation and cytotoxic T lymphocyte activation. By tailoring oxidative stress to exploit tumor-specific vulnerabilities, these approaches effectively overcome drug resistance and improve therapeutic outcomes, presenting significant potential for patient-specific ATP-targeted cancer therapies.

## 4. Increasing eATP for tumor therapy

As a crucial signaling molecule, eATP exerts its influence on various cellular components within the TME by interacting with multiple receptors 3, 5, 113. In tumor cells, the presence of eATP induces alterations in plasma membrane permeability and disruptions in intracellular electrolyte balance, leading to a halt in the S Phase of the cell cycle 114. Consequently, this mechanism effectively impedes the growth and proliferation of tumor cells 114. In the case of immune system cells, eATP facilitates the release of anti-inflammatory factors, such as interleukin-1β (IL-1β) and tumor necrosis factor (TNF), through the activation of DCs. This activation not only enhances antigen presentation in tumors but also contributes to CD8^+^ T lymphocyte response and supports an effective anti-tumor immune response 19. In the TME, the concerted action of CD39 and CD73 enzymes facilitates the degradation of eATP to ADO, thereby transforming the inflammatory milieu mediated by eATP into an immunosuppressive state governed by ADO 3, 41. Excessive ADO production has been shown to impede the immune response against tumors while concurrently facilitating tumor cell proliferation, angiogenesis, and metastasis 115, 116. Generally, the main strategies to increase eATP for bolstering anti-tumor immunity involve delivering exogenous ATP into and ATP release from the TME, as well as inhibiting ATP degradation within the TME.

### 4.1 Delivering exogenous ATP into the TME

Multiple strategies have been developed to deliver exogenous ATP into the TME to inhibit tumor cell proliferation and activate antitumor immune responses 114, 117. A solution of albumin nanoparticles (ANPs) coated with erythrocyte membranes has been proposed for effective loading, delivery, and release of ATP into the TME. The results of experiments at the cellular level have shown that both erythrocyte membrane-coated ANPs and ANPs control the release of ATP for up to 5 days. Coating the erythrocyte membrane can further increase the time for nanoparticle internalization, which is conducive to the extracellular release of ATP 114.

In addition to the controlled release of ATP into the TME, a drug delivery system was designed for the responsive release of ATP 117. The researchers employed a pH-responsive nanoplatform to achieve the targeted delivery of ATP using chitosan-coated mesoporous hydroxyapatite 117. The abundance of ionized amino groups in Chitosan provides potential sites for pH sensitivity. The nanoporous structure of mesoporous hydroxyapatite enhances the loading capacity of ATP and impedes its early release. The drug release kinetics experiment demonstrated spatiotemporal control over ATP release, as it occurred in a time-dependent manner and reached a stable state after 24 h of incubation. Experiments conducted at pH 7.2 and 4.2, revealed that the release of ATP was spatially regulated with sensitivity to pH levels 117.

### 4.2 Promoting ATP release to the TME

**Figure 6:**
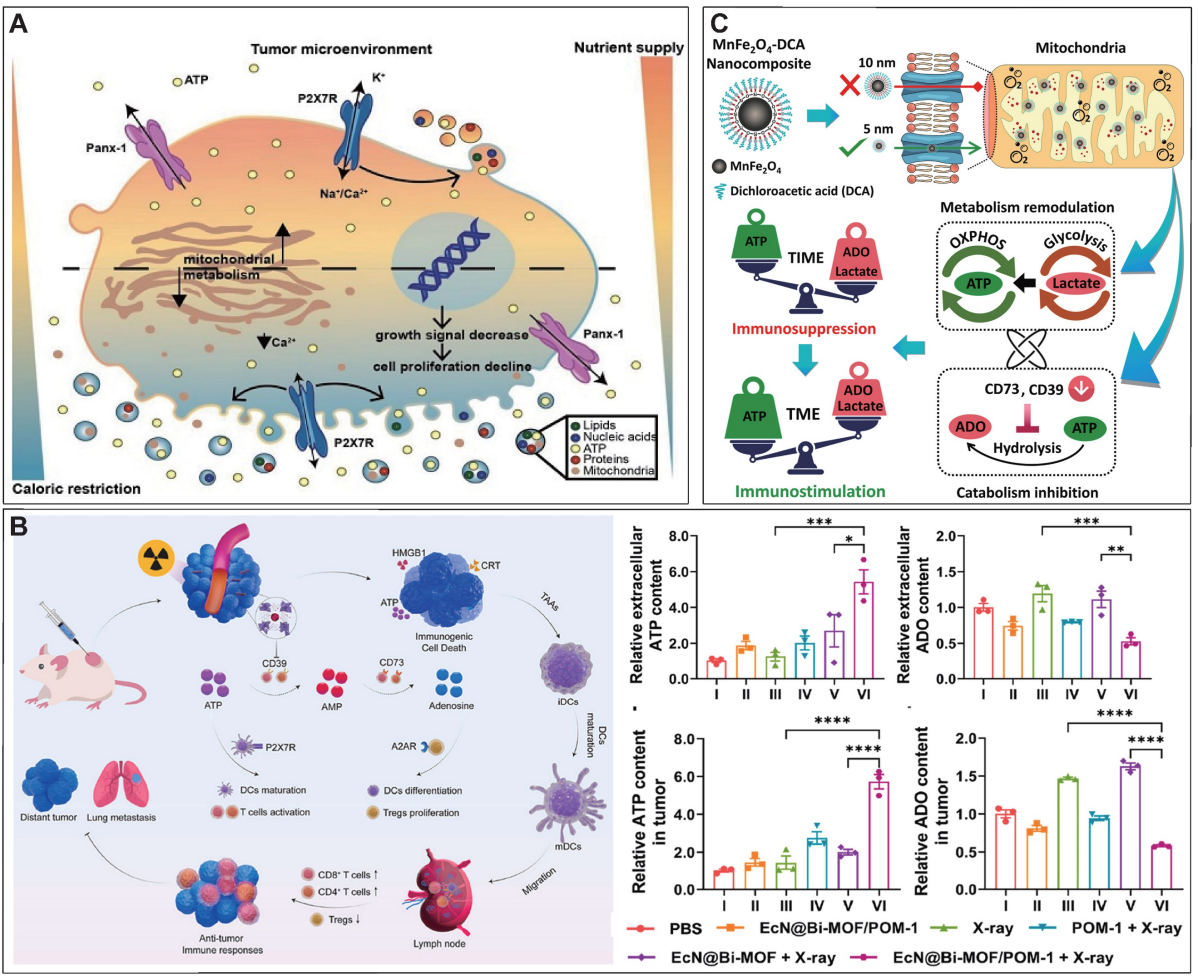
** Nanotechnology-enhanced promotion of eATP.** (A) Nutrient deprivation increases eATP release and ultimately promotes anti-tumor immune response. Adapted with permission from 121, copyright 2022 Theranostics. (B) EcN@Bi-MOF enhances radiation-induced ICD, triggering ATP release from tumor cells while inhibiting eATP degradation. Adapted with permission from 134, copyright 2024 Elsevier. (C) MnFe_2_O_4_ NP conjugated with dichloroacetic acid significantly down-regulated expression of CD39 and CD73, limiting eATP degradation. Adapted with permission from 139, copyright 2022 Elsevier.

In addition to directly delivering exogenous ATP into the TME, strategies have been developed to trigger ATP release from tumor cells in multiple ways 3, 118-120. Caloric restriction has been demonstrated to promote the efficiency of chemotherapy, which is largely attributed to the accumulation of eATP in the TME caused by caloric restriction 3, 120. Specifically, caloric restriction was found to significantly impair oxidative phosphorylation and cause mitochondrial fragmentation in tumor cells, accompanied by a P2X7-dependent release of ATP-loaded, mitochondria-containing microvesicles as well as naked mitochondria 121. Thus, caloric restriction represents a simple but efficient way to promote ATP release into the TME (Figure 6A).

ICD is a form of regulatory cell death that occurs during chemotherapy, radiotherapy, oncolytic virus-mediated therapy, PDT, and PTT, and can activate the adaptive immune response of immunocompetent hosts 122. Dying or stressed tumor cells release molecules called damage-associated molecular patterns (DAMP) that act as adjuvants or danger signals to the immune system 123, 124. ATP, as a main component of DAMP, can be released by dying tumor cells under the different treatment methods 3, 125. eATP-dependent P2X7R stimulation, followed by the release of IL-1β through the activation of NLRP3 inflammatory bodies, stimulates the activation of DCs and presents antigens to CD4^+^ and CD8^+^T lymphocytes, promoting anti-tumor immune response 126. An ICD amplifier that assembles DOX, Fe^2+^, and exogenous ATP has been developed, in which DOX-mediated chemotherapy and Fe^2+^ mediated CDT synergistically induced the release of DAMP by tumor cells 127. Moreover, exogenous ATP can also be delivered into the TME to augment ICD 127. The authors showed that this ICD amplifier with exogenous ATP delivery can activate the immune response to the primary tumor and can also activate a strong abdominal effect on the distal tumor 127. In addition, multiple biomaterial-based strategies were conducted for inducing ICD and trigger the release of ATP for boosting antitumor immune response 128, 129.

### 4.3 Inhibiting the degradation of eATP

The eATP present in the TME can undergo enzymatic degradation mediated by CD39 and CD73, resulting in the generation of ADO, which serves as a crucial immunosuppressive molecule within the TME 41, 113. Therefore, the blockade of eATP degradation through CD39 antibody targeting can enhance the accumulation of eATP within the TME, thereby inhibiting ADO-mediated immunosuppression 130. Researchers have developed a kind of nanoparticles encapsulating the photosensitizer and CD39 inhibitor ARL67156, where, upon laser irradiation, nanoparticle-produced ROS induce ATP release from tumor cells and trigger the cleavage of nanoparticle and the release of ARL67156 from the nanoparticles 131. The released exonuclease inhibitor inhibits dfdthe degradation of eATP to ADO by CD39, thus promoting the anti-tumor immune response 131. Besides CD39 inhibitor, CD73 inhibitor was also incorporated into a nanosystem for interfering ATP-Adenosine Axis and amplifying ICD, which was demonstrated to eventually priming antitumor immune response 132.

AB598, a CD39 inhibitory antibody, provides a promising approach to preserving eATP levels in the TME 133. By targeting CD39 enzymatic activity, AB598 enhances immune activation while reducing immunosuppression in solid tumors. It synergizes with chemotherapy-induced ATP release, amplifying anti-tumor immune responses. Preclinical studies demonstrated significant tumor suppression and excellent safety profiles, highlighting its potential as a candidate for combination immunotherapies. This example further validates the therapeutic utility of targeting CD39 to modulate the ATP-Adenosine axis and strengthen anti-tumor immunity.

A novel bacteria-based biohybrid therapy has been developed to enhance radiotherapy (RT)-triggered antitumor immunity by inhibiting eATP degradation within the TME 134. The biohybrid, termed EcN@Bi-MOF, combines *Escherichia coli* Nissle 1917 (EcN) as a delivery platform, a bismuth-based metal-organic framework (Bi-MOF) as a radiosensitizer, and the CD39 inhibitor POM-1 to preserve eATP levels. Mechanistically, Bi-MOF enhances radiation-induced DNA damage and ICD, while POM-1 prevents ATP degradation into immunosuppressive ADO, sustaining pro-inflammatory ATP signaling (Figure 6B). Preclinical studies demonstrated that EcN@Bi-MOF amplifies ICD markers, promotes dendritic cell maturation, and activates CD8+ T cells, leading to robust systemic immune responses, including the abscopal effect. Notably, this biohybrid therapy significantly inhibited tumor growth, improved survival rates, and reduced regulatory T-cell infiltration without systemic toxicity, underscoring its potential as a safe and effective strategy to remodel the TME and improve RT outcomes.

In solid tumors, the upregulation of CD39 and CD73 can be induced by the hypoxia status, via a transcriptional mechanism that involves hypoxia inducible factor 1 subunit alpha (HIF-1α) 3, 135, 136. Thus, inhibiting the degradation of eATP can also be achieved by alleviating the hypoxia status or blocking the HIF-1α related signal pathway. Our research group developed a calcium phosphate-reinforced iron-based metal-organic frameworks (CaP@Fe-MOFs), in which the Fe moieties undergo Fenton-like reaction and produce O_2_, thus inhibiting the HIF-1α mediated upregulation of CD39 and CD73 137. Thus, the catabolism from ATP to ADO is attenuated, thereby elevating eATP levels, reducing ADO levels, promoting antitumor immune response including promoting dendritic cell maturation, and augmenting cytotoxic T lymphocyte response 137. In addition to alleviating hypoxia for inhibiting HIF-1α mediated upregulation of CD39 and CD73, dichloroacetic acid was shown to block the HIF-1α-CD39/CD73 pathway 138. Dichloroacetic acid was incorporated into a MnFe_2_O_4_ nanocomposites to realize targeted delivery into tumor cells and decrease the eATP catabolism into ADO. In addition, the Fe moieties of this nanocomposites can also produce O_2_ to further downregulate CD39 and CD73 and inhibit the degradation of eATP (Figure 6C) 139.

There are many other therapeutic methods targeting CD39 and/or CD73, including small-molecule inhibitors and antibodies, which have been comprehensively discussed elsewhere 41, 113. However, the complete blockade of CD39 may have unexpected adverse effects. This may be related to CD39's involvement in the regulation of thrombosis, cell adhesion, and other potential physiological events 140.

## 5. ATP responsive drug release for tumor diagnosis and therapy

Drug delivery systems, which use nanoscale components as diagnostic tools or deliver therapeutic drugs to specific sites to enhance therapeutic efficacy and avoid “off-target” effects, are a rapidly evolving field of science 141-143. Because of the heightened glycolytic activity, the ATP level in the TME was significantly higher than that in non-tumor cells, which offers an excellent opportunity to design ATP-responsive drug release platform 2. Here, we comprehensively discuss recent progress in ATP-responsive drug delivery systems.

**Figure 7:**
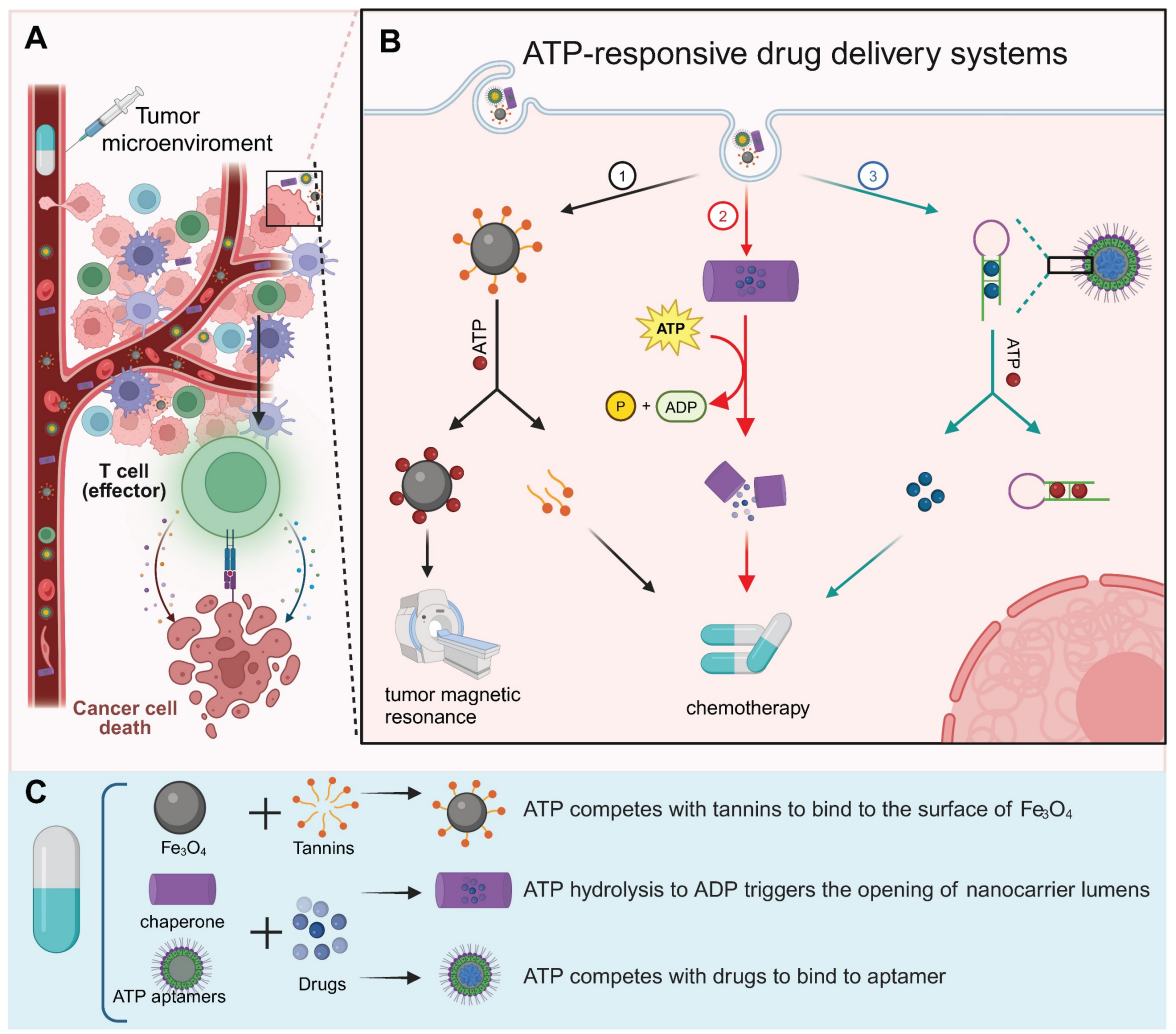
** ATP-responsive drug delivery systems.** (A) ATP-responsive drug delivery system has been extensively studied for cancer diagnosis and therapy. (B) ATP-responsive drug delivery systems fall into three categories based on mechanism: (1) ATP competes with antitumor drugs for binding to the nanoplatform, leading to the release of drugs for tumor therapy; (2) energy release by ATP triggers disintegration of drug delivery cargo, releasing the drug; (3) ATP binds to an aptamer, resulting in NP disintegration and drug release. (C) Fabrication of three representative types of ATP-responsive NPs: tannin-loaded Fe_3_O_4_ NP, barrel-shaped nanocarrier, and aptamers-based NP. Created with BioRender.com. (See the main text for further explanation of these strategies.)

**Figure 8:**
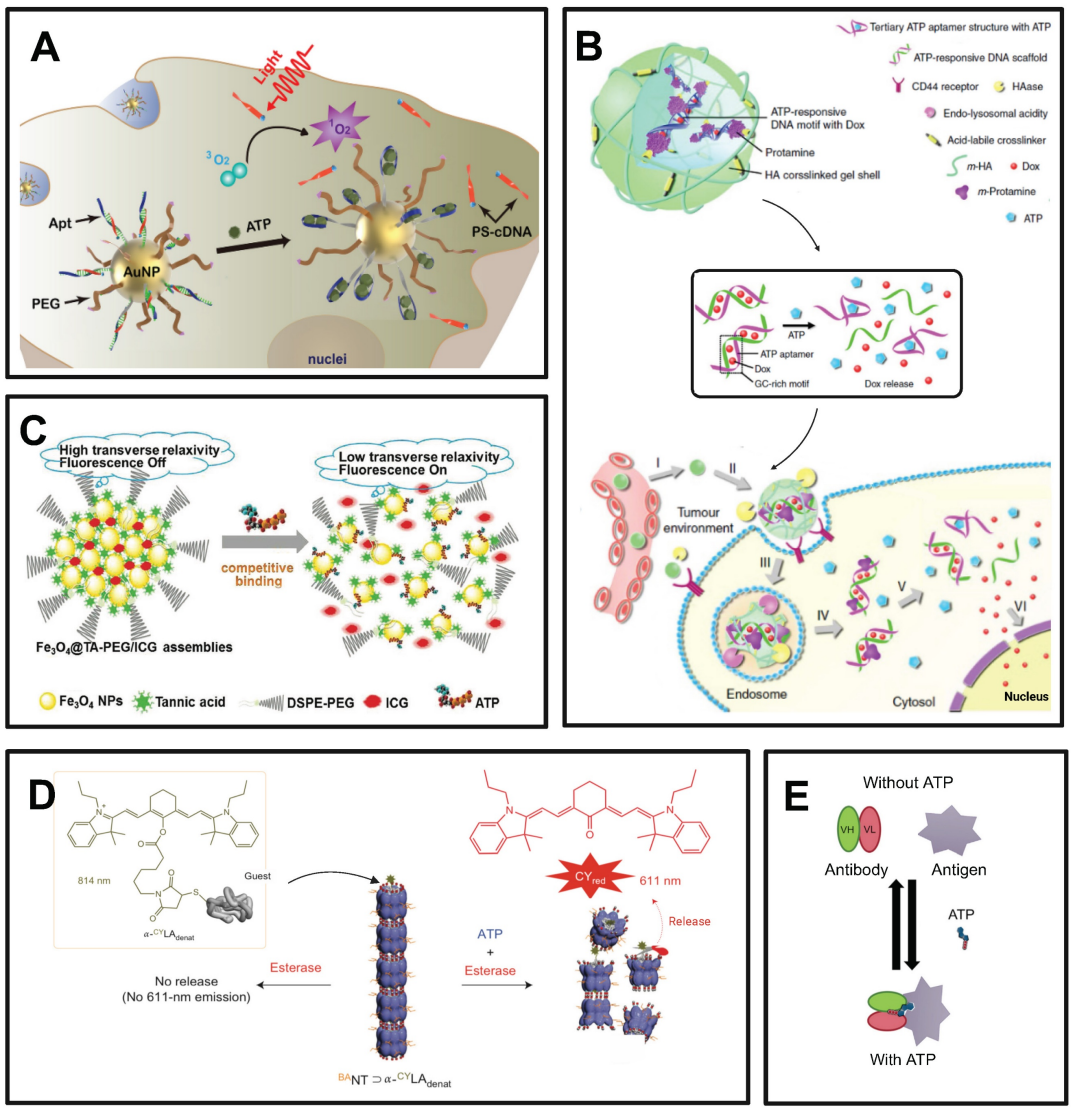
**Examples of ATP-responsive drug delivery nanosystems.** (A) Ligation of ATP and aptamers leads to the disintegration of nanoparticles and the release of photosensitizers, reducing the toxic side effects compared to photodynamic therapy. Adapted with permission from 152, copyright 2020 Springer Nature. (B) NP based on ATP-aptamer selectively releasees DOX through a complex structure transformation when interacting with ATP. Adapted with permission from 153, copyright 2014 Springer Nature. (C) ATP competing with tannins to bind to the surface of Fe_3_O_4_ leads to structure damage of nanoparticle and release of tannins. Adapted with permission from 146, copyright 2017 John Wiley and Sons. (D) iATP induces conformational changes of barrel-shaped protein-based nanocarrier, releasing guest molecules. Adapted with permission from 147, copyright 2013 Springer Nature. (E) Switch antibody binds to target antigen only in the presence of eATP, inhibiting tumor growth with minimal systemic toxicity. Adapted with permission from 148, copyright 2020 Elsevier.

The ATP aptamer, derived through *in vitro* screening technology based on the structure of ATP, exhibits strong recognition of ATP and high affinity 144. The aptamer possesses notable advantages such as specificity, facile synthesis *in vitro*, and modifiability (Figure 7). A diverse range of materials has been employed in the design of aptamer-based ATP response systems, and nanosystems derived from different materials frequently exhibit distinctive merits (Figure 8A and 8B). The details of the process have been extensively reviewed elsewhere 145. Thus, we will focus on the other ATP-responsive drug delivery systems.

In addition to ATP aptamers, there are several alternative approaches to ATP-responsive drug release systems. Plant polyphenols encompass a variety of catechol groups that can coordinate with various metal ions 146. Researchers have used tannins and Fe_3_O_4_ nanoparticles as models to construct polyphenol-inspired intelligent magnetic component 146. Due to the competitive binding of ATP and tannins to the surface of Fe_3_O_4_, the nanosystems comprising intelligent magnetic components demonstrate ATP-responsive characteristics (Figure 8C) 146. In addition, these magnetic components can be employed for tumor-specific and highly sensitive magnetic resonance imaging by specifically disassembling ATP-induced changes that result in enhanced lateral relaxation and efficient conduction of fluorescence signals.

It has been reported that a developed protein-based nanocarrier consists of multiple barrel-shaped chaperone units that protect guest molecules in the lumen from degradation by coordinating with Mg^2+^ assembly into a tubular structure (Figure 8D) 147. The mechanism of the ATP response is due to the symbiotic changes caused by the hydrolysis of ATP to ADP, resulting in the opening and closing of the lumen. Ligation between barrel chaperone units is based on non-covalent bonding formed at nanoscale biological interfaces. This non-covalent bond can be cleaved by mechanical forces resulting from conformational changes in the subunits that constitute the protein. This conformational change is driven by ATP and its analogs and is dependent on the concentration of ATP. Moreover, some studies have reported that antibodies bind to antigens only in the presence of ATP. The high concentration of eATP in the TME serves as a switch for mediating the binding between the antibody and antigen in the TME, which could largely avoid de-tumor toxicity (Figure 8E) 148.

ATP was also demonstrated to competitively displace and disrupt the coordination bond, thereby reactivating the prodrug for the purpose of tumor therapy 149. Specifically, Celastrol, a naturally occurring bioactive compound with significant tumor-eradication potential, is constrained by its limited aqueous stability, diminished bioavailability, and potential off-target effects 149. To address these limitations, the coordination of Fe (III) with Celastrol was employed to synthesize an inert Celastrol-Fe chelate prodrug, which is anticipated to mitigate toxicity to non-target tissues. Notably, within the TME, the elevated ATP levels are capable of competitively displacing the coordination bond, thereby triggering the release of the bioactive Celastrol. The efficacy of this ATP-responsive drug delivery system has been corroborated in both cell-derived xenograft and patient-derived xenograft models, underscoring its potential in targeted cancer therapy 149.

ATP-responsive nanoparticles have demonstrated the potential to achieve precise drug release by leveraging the elevated ATP concentrations within tumor cells 145. However, a critical gap in knowledge remains regarding their specificity across various cancer cell types. This uncertainty primarily arises from tumor heterogeneity, which encompasses variations in ATP concentration and distribution. For instance, tumors with highly glycolytic phenotypes, such as glioblastoma and breast cancer, often exhibit significantly higher ATP levels compared to cancers with lower metabolic states 150, 151. These differences can profoundly influence the performance of ATP-responsive nanoparticles. However, the ATP levels across different cancer types and within distinct regions of tumors have been scarcely investigated, despite their significant impact on the therapeutic efficacy of ATP-responsive nanoparticles. Therefore, comprehensively mapping the spatial distribution of ATP levels within tumors is crucial for a deeper understanding of the delivery characteristics of ATP-responsive systems in tumors. Additionally, identifying suitable tumor types for ATP-responsive delivery systems necessitates the detection of ATP level variations across different tumor types and the evaluation of the delivery efficiency of ATP-responsive systems in these diverse contexts.

## 6. ATP detection for tumor diagnosis

**Figure 9:**
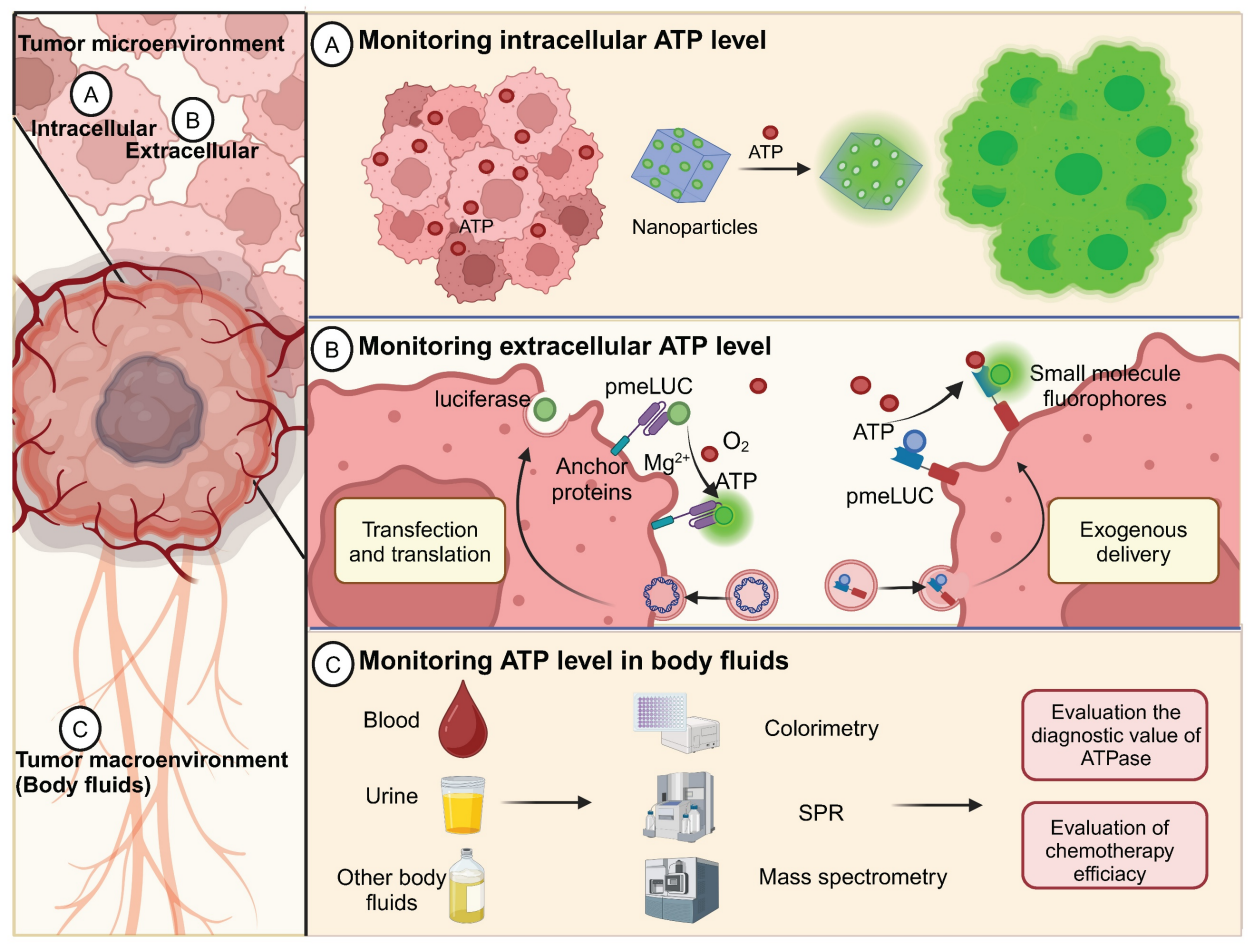
** ATP detection for cancer diagnosis and guided tumor therapy.** (A) In iATP detection, ATP-responsive imaging distinguishes cancer cells from normal cells. (B) In eATP detection in the TME, plasma membrane luciferase (pmeLUC) is used for eATP imaging in TME and for monitoring spatiotemporal dynamics of eATP 1, 158, 159. (C) In body fluids, ATP levels are measured to analyze the level of ATPase as a diagnostic indicator of tumor stage and chemosensitivity. Created with BioRender.com.

**Figure 10:**
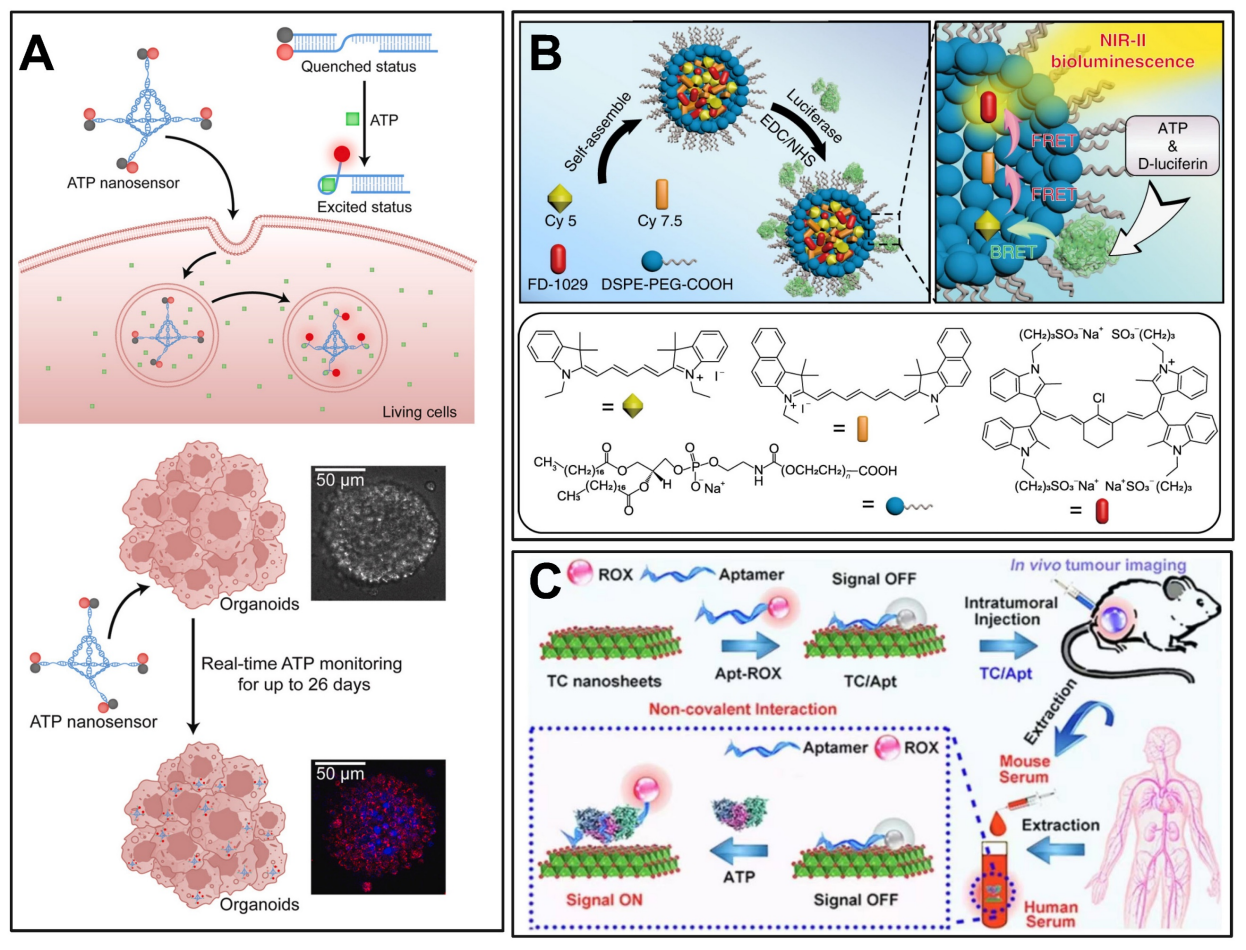
** Examples of ATP detection for cancer diagnosis.** (A) Schematic diagram of ATP detection nanosystems based on DNA tetrahedral framework, primarily used for monitoring iATP. Adapted with permission from 162, copyright 2023 Elsevier. (B) A NIR-II bioluminescence probe integrating cyanide resonance energy transfer and fluorescence resonance energy transfer, using ATP-responsive imaging for tumor diagnosis. Adapted with permission from 156, copyright 2020 Springer Nature. (C) Schematic diagram of ATP monitoring nanosystems based on ATP aptamers predominately for ATP detection *in vitro* and in body fluids. Adapted with permission from 188, copyright 2021 Springer Nature.

The versatile role of ATP as a multifunctional nucleic acid triphosphate extends beyond its function as a primary energy-storage molecule in living organisms. ATP also serves as an extracellular signaling molecule involved in regulating cell metabolism and biochemical signaling pathways, making its abnormality closely related to the progression and therapeutic strategies of cancer 113, 154. Therefore, monitoring the spatiotemporal dynamics of ATP in tumor cells 155, the TME 156, and body fluids 157 in patients with tumors is critical, and much research effort has been dedicated to this area (Figure 9, 10).

### 6.1 Monitoring intracellular ATP

ATP is the main source of energy for an organism and is overexpressed in tumors; its accumulation enhances the proliferation and migration of cancer cells. Therefore, measuring iATP levels may be helpful in the pathological biopsy of tumors. In a recent study, a two-channel nanoprobe was used for the sequential detection of ATP and peroxynitrite to distinguish between normal and cancer cells 155.

Nanocarriers, such as liposome and exosome coatings, possess a structural resemblance to cell membranes 160. This similarity allows the cell to easily internalize the loaded ATP-detecting probe, thereby efficiently detecting iATP. A liposome carrying fluorescent ATP aptamers was designed to be engulfed by the cells 161. Upon cellular entry, ATP molecules bind to the aptamers and induce a conformational change that leads to the formation of a p-aniline-ATP complex. This new conformation interacted closely with the guanine nucleobase, resulting in strong fluorescence quenching via intramolecular fluorescence resonance energy transfer. Notably, a robust linear relationship exists between the extent of quenching and the ATP concentration, enabling the accurate determination of ATP levels 161. The authors of the study also used exogenous oligomycin to modulate the inhibition of ATP synthetase and to monitor the dynamic assessment of ATP production 161.

DNA tetrahedral framework-based nanosensors represent a novel strategy for long-term ATP monitoring in complex biological models. A study by Zhang *et al.* developed a DNA tetrahedral framework (TDF) conjugated with ATP-specific aptamer strands (TDF-AS) for real-time ATP tracking in human cancer organoids over 26 days (Figure 10A) 162. The TDF scaffold ensured high biocompatibility and lysosomal escape, while ATP binding triggered fluorescence via FRET between Cy3 and BHQ2. This approach enabled non-destructive monitoring of organoid growth and drug responses (e.g., paclitaxel-induced apoptosis) with minimal cytotoxicity. The study highlighted the potential of DNA nanotechnology for longitudinal ATP analysis in fragile 3D organoid models, addressing limitations of single-time-point detection methods.

Zeolitic imidazolate framework 8 (ZIF-8), a widely studied carrier for drug delivery, was fabricated using Zn^2+^ and imidazole linkers 163. A ZIF-8 carrying rhodamine B (RhB) was developed, in which the fluorescence of RhB was quenched by the shielding effect of ZIF-8. However, in the presence of ATP, RhB fluorescence occurs because the disintegration of ZIF-8 is caused by competitive coordination between ATP and Zn^2+^, which provides an efficient tool for ATP detection 164. In addition, cancer cell-derived exosome membranes were utilized to encapsulate this nanodevice, which further improved the cellular uptake efficiency for better monitoring of iATP 164.

A straightforward approach for detecting ATP levels is the insertion of a single glass nanopipette-based nanopore (G-nanopore) into cells 165. The Three-thiol-modified DNA (probe 1) and 5-thiol-modified DNA (probe 2) contain fragments that bind to the ATP aptamer, which is further autonomously assembled as Au nanoparticle self-assemblies (NPSAs). In the presence of ATP, the NSPAs were gradually disassembled because of the stronger combination of ATP and aptamers, expanding the effective aperture and increasing the ion current to 500 mV. Moreover, experiments show that the ATP concentration has a good linear relationship with the current change ratio at 500 mV 165.

### 6.2 *In vivo* ATP imaging in TME

There is a fundamental interdependence between ATP dynamics and physiology that occurs inside and outside tumor cells 4. Thus, we need a way to investigate the temporal and spatial dynamic changes in ATP in living specimens from the subcellular to the biological level in patients with tumors. Visualization of ATP can intuitively distinguish between tumors and healthy tissues and can accurately guide tumor treatment and avoid damage to healthy tissues.

#### 6.2.1 ATP imaging for tumor diagnosis

Bioluminescence has a wide range of applications in ATP imaging 166-168. When conventional bioluminescence imaging is performed *in vivo*, strong tissue absorption and scattering at short wavelengths limit the improvements in sensitivity and deep-tissue resolution 169. To address these issues, researchers have achieved a higher spatial resolution in deep tissues using optical imaging in the second near-infrared region (NIR-II) 156. An NIR-II bioluminescence probe integrating cyanidation resonance energy transfer and fluorescence resonance energy transfer was developed and successfully applied to vascular and lymphatic imaging of mice (Figure 10B) 156. The spatial resolution of the probe was approximately 1.5 times higher than that of conventional bioluminescence imaging, and the recognition rate of tumor metastasis was 83.4 times higher than that of normal tissue by utilizing the ATP response characteristics. *In situ* ATP-mediated tumor-tracking experiments in nude mice showed that, compared with conventional bioluminescence imaging methods, the tumor/normal ratio of NIR-II fluorescence imaging can be enhanced by 30%, which has an excellent ability to distinguish between tumor and healthy tissues 156.

In addition, ATP imaging can be combined with other iconic biological signals to further improve the tumor-to-normal tissue ratio, which is conducive for tumor diagnosis. For example, glutathione 170, 171, H_2_O_2_ 172, and miRNAs abnormally expressed in cancer 173 have been introduced for this purpose.

#### 6.2.2 Optimization of ATP imaging strategies

*In vivo* ATP imaging could enable an understanding of changes in ATP levels in different subcellular regions, providing key details regarding cell physiology and metabolic regulation 158, 159, 174. PmeLUC is a chimeric lecithin folate receptor whose chimeric proteins are targeted and retained on the plasma membrane, where the luciferase converts d-luciferin to oxidized luciferin in the presence of ATP and is catalyzed by magnesium ions, producing a light signal that can be captured by sensitive detectors *in vivo* or *in vitro* 158. As early as 2008, researchers generate pmeLUC probe stably expressing cells that act as ATP concentration sensors in the TME 1. However, during the experiments, it was found that the strong luminescence signal was not only related to the concentration of ATP but was also affected by the temporal and spatial uniformity of the pmeLUC probe distribution, the exogenous transport of luciferin, and the amount of molecular oxygen.

Further research solved these problems: a comprehensive set of ATP-binding protein cysteine-substituted mutants was developed and labeled with a small-molecule fluorophore Cy3 at the introduced cysteine residue, with non-toxic subunits added to botulinum neurotoxin for binding to the neuronal plasma membrane 159. The resulting Cy3-labeled glutamine-105 mutant (Q105C-Cy3; designated ATPOS) complex was attached to neuronal cell membranes in the cerebral cortex of living mice, showing concentric propagation waves caused by eATP under electrical stimulation. Using two-color ratio imaging, the ATPOS complex displayed significant fluorescence changes in the red channel (Cy3), but no changes in the green channel (Alexa488). This allows for the measurement of ATP concentration by comparing the red and green fluorescence ratios, which reduces sensor concentration variations and motion artifacts 159.

### 6.3 Monitoring of ATP levels in body fluids

Liquid biopsy for tumor diagnosis has long been the focus of scientific research and has recently experienced a boom 175-177. Significant efforts have been made to identify markers in liquid biopsies for early detection, treatment stratification, and monitoring for cancer recurrence 176, 178. Serum ATPase has been demonstrated to be a promising diagnostic marker compared with traditional hematological tumor biomarkers 179, 180. In the plasma of prostate cancer patients, ATPase-mediated ATP hydrolysis is at a high level, which subsequently produces a large amount of ADO and favors the progression of tumors 157. Therefore, monitoring the dynamic changes in ATP levels in body fluids may indirectly indicate ATPase activity. In addition, the ADO level can also be reflected by dynamic changes in ATP concentration, which helps evaluate the immunosuppressive status of tumors. Currently, various methods have been applied to detect ATP in body fluids, such as colorimetry 181, electrochemical methods 182, fluorescent labeling 63, mass spectrometry 183, and surface plasmon resonance (SPR) 184.

SPR is a sensitive surface spectroscopy technique by which refractive index changes on the surface of gold films in the range of 200 nm are monitored in real time 185; the results are highly sensitive to refractive index changes caused by the binding or dissociation of biomolecules on the surface 185. Researchers have detected ATP by using silver ions to induce cytosine (C) single-stranded DNA probe configuration changes based on SPR 186. This DNA probe has dual recognition sites for ATP and silver ions: an ATP aptamer and five cytosines located at either end of the probe. In the absence of ATP, the probe forms a rigid hairpin C-Ag ^+^-C structure, generating a large SPR signal. In the presence of ATP, aptamers interact with ATP to form a three-dimensional structure; the configuration change of the silver ion-induced probe is small, and the SPR signal is small. The difference in the SPR signal before and after the probe interaction with ATP is proportional to the concentration of the analyte, which excluded the effect of SPR signal from non-specific absorption 186.

Fluorescence methods are widely used for ATP detection 187. The ATP concentration can be obtained using a linear relationship between the changes in fluorescence intensity and ATP concentration. The novel CuO-modified Zr-MOF nanocomposite acts as a nano platform for H_2_O_2_ and ATP fluorescence detection in the presence of terephthalic acid (TA). The CuO nanoparticles exhibited peroxidase activity and catalyzed the reaction of TA and H_2_O_2_ to produce fluorescent TAOH. However, in the presence of ATP, the catalytic reaction of CuO with H_2_O_2_ is hindered by the interaction of ATP with Zr^4+^ and CuO, resulting in a decrease in the fluorescence intensity of TAOH at 425 nm 187. ATP concentration has a good linear relationship with fluorescence intensity between 0.001and 30 µM. However, the authors reported that the sensitivity of ATP detection decreased at high concentrations, which is likely related to the saturation of the surface sites in nano systems 187. Another fluorescence-based detection method uses titanium carbide (TC) nanosheets modified with ATP aptamers (TC/Apt) (Figure 10C) 188. This technique utilizes Fluorescence Resonance Energy Transfer, where TC nanosheets act as quenchers of fluorescence, which is restored upon binding to ATP. The TC/Apt probes demonstrate a wide detection range (1 μM - 1.5 mM ATP) and a low detection limit (0.2 μM), making them highly sensitive for ATP monitoring. The probes successfully detected ATP in mouse serum and human serum and were able to visualize ATP changes in living cells and tumor models.

Similarly, ATP concentration can be obtained using the relationship between the control voltage signal intensity and ATP concentration in sensitive electrochemical detection. In one study, an electrochemical biosensor was developed based on a porous Fe_3_O_4_@covalent organic framework with AuNPs confined within uniform channels 189. The authors of this study found that the hydrophobic porous nanochannels established a distinct and stable microenvironment, conferring exceptional electrocatalytic performance in the reduction of 4-nitrophenol (4-NP). Single-stranded DNA (S_0_) immobilized on the surface of the nanoplatform triggers a branched-chain hybridization reaction by interacting with the conversion product of ATP (S_1_). This reaction was found to impede the electron transfer between 4-NP and AuNPs, enabling the sensitive detection of ATP 189.

In addition to the various detection methods mentioned above, a number of other excellent strategies have been proposed to improve detection efficiency through pre-concentration 190 and matrixing 183. Some researchers have adopted ingenious methods for detecting ATP through pre-concentration. Nanocomposites composed of mixed metal hydroxides and magnetic nanoparticles act as efficient green extractants and peroxidase catalysts to adsorb trace ATP from the sample solution to the surface of the nanoparticles to complete the pre-concentration 190. The concentration of ATP affects the fluorescence intensity generated by the reaction of H_2_O_2_ and o-phenylenediamine catalyzed by magnetic nanoparticles peroxidase. ATP adsorbs onto the surface of the nanomaterial, thereby reducing its active area and decreasing the production of fluorescent matter. Thus, ATP detection was achieved. Similarly, some researchers have used ATP aptamer-gold nanoparticles to adsorb ATP as a selection probe and measured ATP by mass spectrometry, which improved the efficiency of the laser ionization matrix 183.

### 6.4 ATP imaging for treatment guidance

ATP imaging can not only be used for the early diagnosis of tumors but can also be used to precisely guide tumor treatment, allowing selective ablation of tumors and reducing side effects 173. In a previous study, a nanosystem consisting of hybrid micellar polymers integrated with nucleoleole-targeted aptamers and ATP aptamers of a Black Hole Quencher 2 (BHQ2)-labeled quenching apparatus was developed to deliver ATP-activating photosensitizers into tumor cells in a targeted manner 173. Injection of the nanosystems into HeLa cells of living mice enabled the acquisition of longitudinal whole-body fluorescence images of mice over time. Experiments in mice have shown that PDT guided by real-time fluorescence imaging is highly effective. After 18 days of treatment, the tumors of the mice were eliminated, and the tissue sections showed significant apoptotic cell populations 191. On this basis, they also made improvements. Replace ATP-activated photosensitizers with miRNA stimulation photosensitizers and realize "early detection and early treatment" of cancer by PDT with a cascade amplification effect 173. In addition, ATP imaging can be used in conjunction with chemotherapy. Near-infrared fluorescent dye (RhI) and DOX were encapsulated in the ZIF-90 framework. As the ZIF framework hydrolyzes because of the ATP response, RhI is released, providing excellent NIR emissions, and the release of DOX allows for controlled chemotherapy drug delivery 192.

## 7. Conclusion

**Figure 11:**
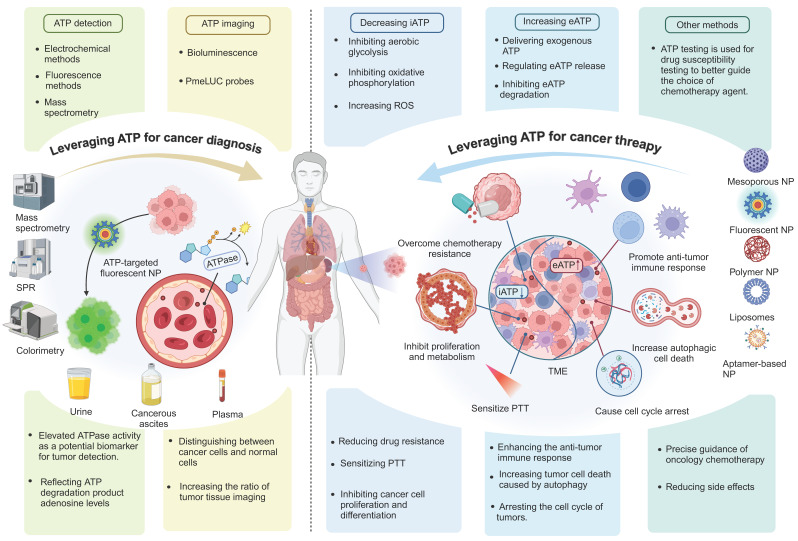
** Summary of ATP-based strategies in cancer theranostics.** ATP can be leveraged for both cancer diagnosis and therapy. Levels of ATPase can be estimated by detecting ATP concentration in urine, ascites, plasma and other materials. Several detection methods are applied such as mass spectrometry, SPR, and colorimetry. Additionally, ATP imaging can be used to distinguish cancer cells from normal cells. In cancer therapy, the dual effect of ATP (tumor promoting and inhibiting) translates to strategies for cancer therapy that are focused on either decreasing iATP or increasing eATP. Decreasing iATP is beneficial to block chemotherapy resistance, inhibit proliferation and metabolism, and sensitize PTT. Increasing eATP can promote anti-tumor immune response, increase autophagic cell death and cause cell cycle arrest. In terms of improved drug delivery, the application of nanoparticles that interact with ATP, compassing mesoporous nanoparticles, fluorescent nanoparticle, polymer nanoparticle, liposomes and aptamer-based nanoparticle, can reduce side effects and improve therapy against tumors. Lastly, ATP detection is used in drug sensitivity tests to better guide the selection of chemotherapy drugs. Created with BioRender.com.

ATP-based cancer diagnosis and treatment strategies have been well developed over the years. Monitoring changes in ATP levels and targeted ATP imaging can be used for the diagnosis of cancer; depletion of iATP can starve tumors, and increasing eATP can promote anti-tumor immune responses and ICD. Here we have given a comprehensive overview of techniques using ATP in both the diagnosis and treatment of cancer (Figure 11). There are multiple strategies for regulating ATP for tumor diagnosis and therapy, and some of these techniques are already in clinical development. The clinical advancement of strategies designed to reduce ATP production for tumor starvation therapy primarily focuses on key targets within energy metabolism pathways. These strategies include inhibitors of GLUTs inhibitors of key enzymes in the glycolytic pathway (such as PKM2), inhibitors of oxidative phosphorylation, and so on 193, 194. For example, Metformin has been confirmed as a promising targeted drug for complex I and, when used in combination with chemotherapy agents, has shown promising results in several clinical trials 195. On the other hand, for modulating ATP metabolism to activate anti-tumor immunity, the primary targets are CD39 and CD73. Currently, multiple CD39 and CD73 inhibitors are in clinical trials for tumor treatment. Notably, the CD73 inhibitor LY3475070 in combination with PD-1 monoclonal antibody has completed phase I clinical trials for advanced solid tumors (NCT04148937), and the CD39 inhibitor IPH5201 in combination with PD-L1 monoclonal antibody and CD73 inhibitor has also completed phase I clinical trials for advanced solid tumors (NCT04261075). However, regarding ATP-responsive drug release for tumor diagnosis and therapy, as well as ATP detection for tumor diagnosis, these remain in the preclinical phase, with no products yet entering clinical trials. And we consider several important issues that need to be addressed, and potential avenues that still need to be explored. First, blocking iATP production decreases ATP efflux into the extracellular space, thereby exacerbating the immunosuppressive status of the TME. Thus, depleting iATP for tumor starvation is a double-edged sword that reminds us to monitor the immune status of the TME when this method is applied. Second, evidence also suggests that some types of tumors would harness eATP for their progression and resistance to therapy 196, 197, which means that delivery of exogenous ATP into the TME may not mediate optimal anticancer effects in some settings, and the strategy of promoting ATP release into the TME from dying tumor cells would be better for this situation. Third, most studies focusing on ATP-based diagnostic and therapeutic strategies for single cancer types have shown promising results. However, the vast majority of these studies have not adequately addressed the issue of tumor heterogeneity. Tumor heterogeneity manifests at three levels: first, there are significant differences among various tumor types 198; second, heterogeneity exists among individuals with the same type of tumor 199; and third, spatial variations occur within a single tumor 200, 201. These heterogeneity factors can lead to substantial fluctuations in ATP concentrations, which in turn may profoundly impact the effectiveness of ATP-based tumor diagnosis and therapy. Tumor heterogeneity thus represents a major challenge for both ATP imaging-based tumor diagnosis and ATP-responsive drug delivery. In addition to highly metabolic tumor cells, some persistently surviving tumor cells exhibit low levels of metabolism and proliferation 202; their reduced metabolic activity results in limited ATP content, thereby restricting the applicability of ATP imaging and ATP-responsive drug delivery. In the strategy of enhancing anti-tumor immunity by increasing eATP, tumor heterogeneity plays a decisive role. In “cold” tumors, which are characterized by weak immune responses and sparse immune cell infiltration, elevating eATP levels may be counterproductive—not only failing to activate an immune response but also potentially stimulating tumor cell proliferation and migration. Therefore, for “cold” tumors, the strategy of elevating eATP to activate anti-tumor immunity should be approached with caution, with a preference for strategies that reduce iATP; In contrast, in “hot” tumors, which exhibit robust immune responses and abundant immune cell infiltration, increasing eATP is expected to significantly enhance anti-tumor immunity. Thus, for “hot” tumors, the eATP-increasing approach should be actively pursued. However, when administering exogenous ATP nanoparticle drugs, it is crucial to engineer these nanoparticles with immune cell-targeting capabilities to ensure that the drugs primarily affect immune cells rather than tumor cells. Despite these and other incognitudes, ATP stands out as a particularly upcoming key for the evolution of tumor diagnosis and therapy. Future research should prioritize several critical areas to improve the efficacy and applicability of ATP-based strategies. The iATP-based tumor starvation therapy may simultaneously reduce the immune-stimulating effects of eATP.

Therefore, balancing iATP depletion with immune modulation remains a key focus, necessitating approaches that integrate iATP-depleting therapies with methods to sustain a pro-inflammatory TME; In the process of increasing eATP to stimulate immunity, optimizing eATP utilization is important, as certain tumors exploit eATP to promote growth and therapeutic resistance. Promoting ATP release from dying tumor cells, rather than delivering exogenous ATP, may help address these challenges; Advancing ATP-responsive drug delivery systems requires efforts in material optimization, scalability enhancement, and rigorous clinical trials to ensure their successful translation into clinical practice; Additionally, refining ATP imaging technologies to achieve higher sensitivity, resolution, and compatibility with multimodal imaging platforms will be crucial; Investigating the role of ATP in cancer stem cells and persistent tumor cells with low metabolic activity may provide new insights and unlock novel therapeutic strategies; Lastly, combination therapies that integrate ATP-based approaches with other treatment modalities, such as immunotherapy or radiotherapy, offer significant potential for synergistic effects and should be explored further. By addressing these research priorities, ATP-based strategies can evolve into more precise, effective, and personalized tools for advancing cancer therapy.

## Figures and Tables

**Figure 1:**
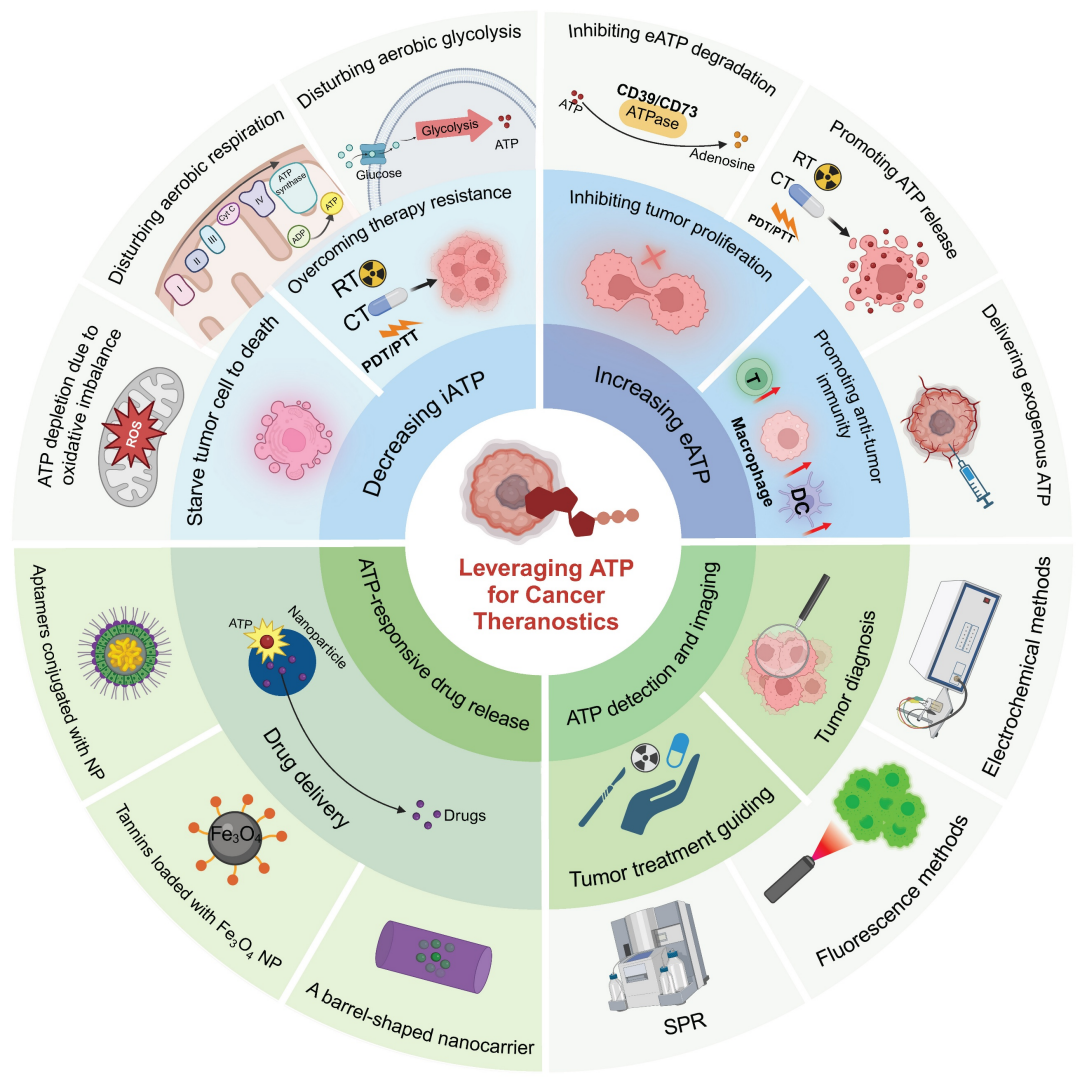
** Illustration of diverse strategies for harnessing ATP in cancer theranostics.** ATP-based tumor theranostics consists of four different approaches: 1) the reduction of iATP levels to induce cancer cell starvation, 2) the elevation of eATP levels to prime antitumor immune responses or inhibit tumor proliferation, 3) ATP-responsive drug delivery, and 4) ATP detection for tumor diagnosis and guided tumor therapy. In the first approach, strategies to decrease iATP levels in tumors are largely based on perturbing mitochondrial energy metabolic processes, including disrupting redox equilibrium and interfering with aerobic glycolysis and respiration. In the second approach, the emphasis is on the maintenance of a high eATP level by delivering exogenous ATP, promoting ATP release, and inhibiting ATP degradation. In the third approach—ATP-responsive drug delivery—nanoparticles interact in a complex way with ATP. Three representative nanoparticles are: aptamer-based nanoparticles, tannins-loaded Fe_3_O_4_ nanoparticles, and barrel-shaped nanocarriers. Lastly, for ATP detection and imaging in tumor diagnosis and guided therapy (including imaging-guided resection as well as sensitivity determination of chemotherapy and radiotherapy), multiple methods, such as electrochemical techniques along with surface plasmon resonance (SPR) and fluorescence methods, are employed. Created with BioRender.com.

**Figure 5:**
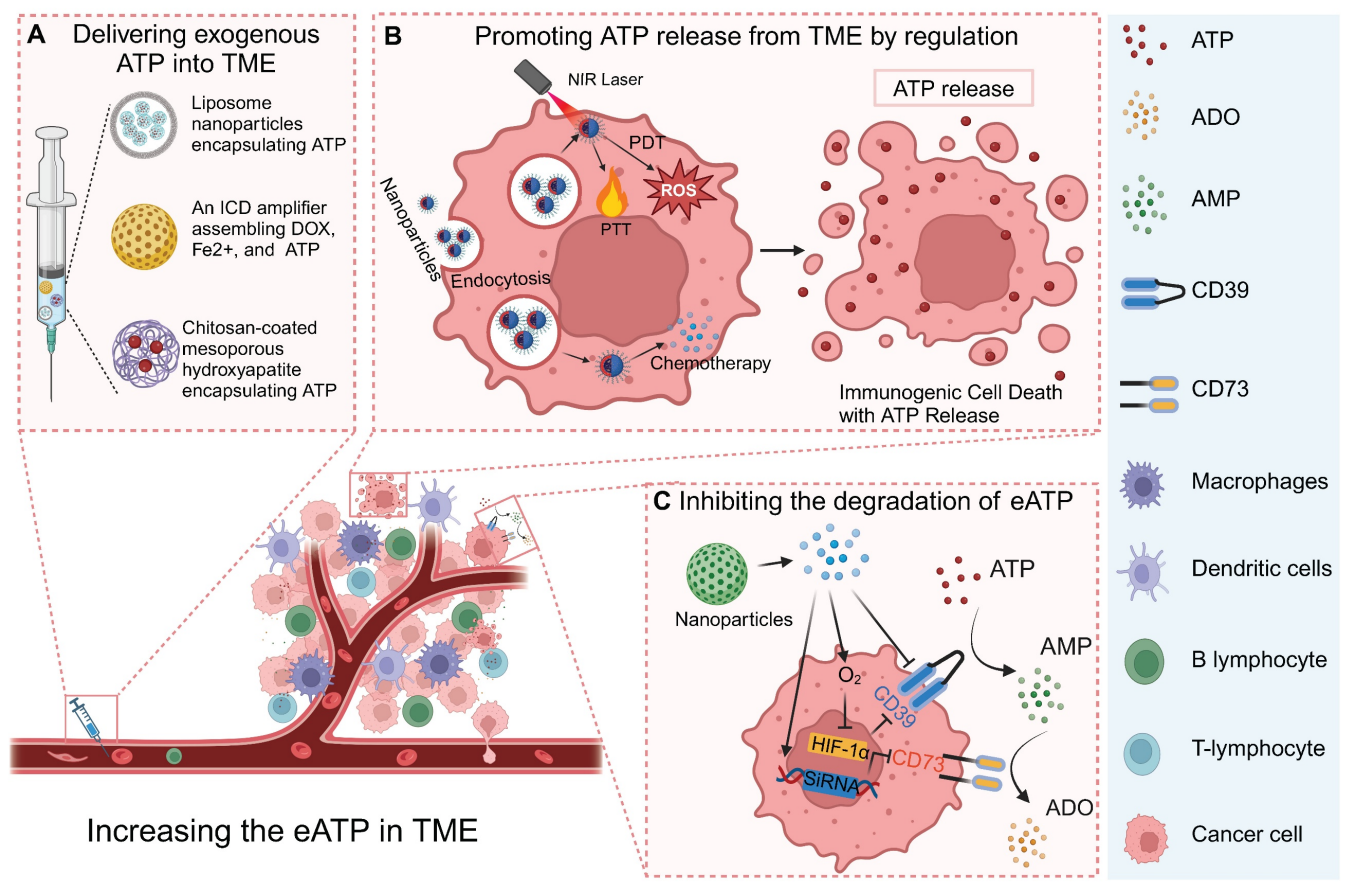
** Nanoparticle (NP) technologies to increase eATP for enhancing anti-tumor immune response.** (A) eATP delivered into TME through nanoparticles such as liposome NPs. (B) NPs induce ATP release caused by ICD. (C) NPs carrying O_2_ or inhibitors of CD39 and CD73 effectively inhibit degradation of ATP, boosting anti-tumor immune response. Created with BioRender.com.

## References

[1] Pellegatti P, Raffaghello L, Bianchi G, Piccardi F, Pistoia V, Di Virgilio F (2008). Increased level of extracellular ATP at tumor sites: in vivo imaging with plasma membrane luciferase. PLoS One.

[2] Di Virgilio F, Sarti AC, Falzoni S, De Marchi E, Adinolfi E (2018). Extracellular ATP and P2 purinergic signalling in the tumour microenvironment. Nat Rev Cancer.

[3] Kepp O, Bezu L, Yamazaki T, Di Virgilio F, Smyth MJ, Kroemer G (2021). ATP and cancer immunosurveillance. EMBO J.

[4] Bergers G, Fendt SM (2021). The metabolism of cancer cells during metastasis. Nat Rev Cancer.

[5] Vultaggio-Poma V, Sarti AC, Di Virgilio F (2020). Extracellular ATP: A Feasible Target for Cancer Therapy. Cells.

[6] Allard B, Allard D, Buisseret L, Stagg J (2020). The adenosine pathway in immuno-oncology. Nat Rev Clin Oncol.

[7] Ruiz-Fernandez de Cordoba B, Martinez-Monge R, Lecanda F (2023). ENPP1 Immunobiology as a Therapeutic Target. Clin Cancer Res.

[8] Yegutkin GG, Boison D (2022). ATP and Adenosine Metabolism in Cancer: Exploitation for Therapeutic Gain. Pharmacol Rev.

[9] Jones CL, Inguva A, Jordan CT (2021). Targeting Energy Metabolism in Cancer Stem Cells: Progress and Challenges in Leukemia and Solid Tumors. Cell Stem Cell.

[10] Alvarez CL, Troncoso MF, Espelt MV (2022). Extracellular ATP and adenosine in tumor microenvironment: Roles in epithelial-mesenchymal transition, cell migration, and invasion. J Cell Physiol.

[11] Di Virgilio F, Vultaggio-Poma V, Tarantini M, Giuliani AL (2024). Overview of the role of purinergic signaling and insights into its role in cancer therapy. Pharmacol Ther.

[12] Vultaggio-Poma V, Di Virgilio F (2022). P2 Receptors: Novel Disease Markers and Metabolic Checkpoints in Immune Cells. Biomolecules.

[13] Burnstock G, Di Virgilio F (2013). Purinergic signalling and cancer. Purinergic Signal.

[14] Burnstock G (2007). Physiology and pathophysiology of purinergic neurotransmission. Physiol Rev.

[15] Adinolfi E, Cirillo M, Woltersdorf R, Falzoni S, Chiozzi P, Pellegatti P (2010). Trophic activity of a naturally occurring truncated isoform of the P2X7 receptor. FASEB J.

[16] Adinolfi E, Callegari MG, Ferrari D, Bolognesi C, Minelli M, Wieckowski MR (2005). Basal activation of the P2X7 ATP receptor elevates mitochondrial calcium and potential, increases cellular ATP levels, and promotes serum-independent growth. Mol Biol Cell.

[17] Amoroso F, Capece M, Rotondo A, Cangelosi D, Ferracin M, Franceschini A (2015). The P2X7 receptor is a key modulator of the PI3K/GSK3beta/VEGF signaling network: evidence in experimental neuroblastoma. Oncogene.

[18] Amoroso F, Falzoni S, Adinolfi E, Ferrari D, Di Virgilio F (2012). The P2X7 receptor is a key modulator of aerobic glycolysis. Cell Death Dis.

[19] Junger WG (2011). Immune cell regulation by autocrine purinergic signalling. Nat Rev Immunol.

[20] Sáez PJ, Vargas P, Shoji KF, Harcha PA, Lennon-Duménil A-M, Sáez JC (2017). ATP promotes the fast migration of dendritic cells through the activity of pannexin 1 channels and P2X7 receptors. Science Signaling.

[21] Shukla S, Dalai P, Agrawal-Rajput R (2024). Metabolic crosstalk: Extracellular ATP and the tumor microenvironment in cancer progression and therapy. Cell Signal.

[22] Ferrari D, La Sala A, Chiozzi P, Morelli A, Falzoni S, Girolomoni G (2000). The P2 purinergic receptors of human dendritic cells: identification and coupling to cytokine release. FASEB J.

[23] Mutini C, Falzoni S, Ferrari D, Chiozzi P, Morelli A, Baricordi OR (1999). Mouse dendritic cells express the P2X7 purinergic receptor: characterization and possible participation in antigen presentation. J Immunol.

[24] Ghiringhelli F, Apetoh L, Tesniere A, Aymeric L, Ma Y, Ortiz C (2009). Activation of the NLRP3 inflammasome in dendritic cells induces IL-1beta-dependent adaptive immunity against tumors. Nat Med.

[25] He Y, Taylor N, Fourgeaud L, Bhattacharya A (2017). The role of microglial P2X7: modulation of cell death and cytokine release. J Neuroinflammation.

[26] Zanin RF, Bergamin LS, Morrone FB, Coutinho-Silva R, de Souza Wyse AT, Battastini AM (2015). Pathological concentrations of homocysteine increases IL-1β production in macrophages in a P2X7, NF-ĸB, and erk-dependent manner. Purinergic Signal.

[27] Barberà-Cremades M, Gómez AI, Baroja-Mazo A, Martínez-Alarcón L, Martínez CM, de Torre-Minguela C (2017). P2X7 Receptor Induces Tumor Necrosis Factor-α Converting Enzyme Activation and Release to Boost TNF-α Production. Front Immunol.

[28] Li XY, Moesta AK, Xiao C, Nakamura K, Casey M, Zhang H (2019). Targeting CD39 in Cancer Reveals an Extracellular ATP- and Inflammasome-Driven Tumor Immunity. Cancer Discov.

[29] Woehrle T, Yip L, Elkhal A, Sumi Y, Chen Y, Yao Y (2010). Pannexin-1 hemichannel-mediated ATP release together with P2X1 and P2X4 receptors regulate T-cell activation at the immune synapse. Blood.

[30] Feng LL, Cai YQ, Zhu MC, Xing LJ, Wang X (2020). The yin and yang functions of extracellular ATP and adenosine in tumor immunity. Cancer Cell Int.

[31] Jacob F, Pérez Novo C, Bachert C, Van Crombruggen K (2013). Purinergic signaling in inflammatory cells: P2 receptor expression, functional effects, and modulation of inflammatory responses. Purinergic Signal.

[32] Salles É M, Menezes MN, Siqueira R, Borges da Silva H, Amaral EP, Castillo-Méndez SI (2017). P2X7 receptor drives Th1 cell differentiation and controls the follicular helper T cell population to protect against Plasmodium chabaudi malaria. PLoS Pathog.

[33] Borges da Silva H, Beura LK, Wang H, Hanse EA, Gore R, Scott MC (2018). The purinergic receptor P2RX7 directs metabolic fitness of long-lived memory CD8(+) T cells. Nature.

[34] Aswad F, Kawamura H, Dennert G (2005). High sensitivity of CD4+CD25+ regulatory T cells to extracellular metabolites nicotinamide adenine dinucleotide and ATP: a role for P2X7 receptors. J Immunol.

[35] Dang EV, Barbi J, Yang HY, Jinasena D, Yu H, Zheng Y (2011). Control of T(H)17/T(reg) balance by hypoxia-inducible factor 1. Cell.

[36] Amoroso F, Capece M, Rotondo A, Cangelosi D, Ferracin M, Franceschini A (2015). The P2X7 receptor is a key modulator of the PI3K/GSK3β/VEGF signaling network: evidence in experimental neuroblastoma. Oncogene.

[37] Yan J, Li XY, Roman Aguilera A, Xiao C, Jacoberger-Foissac C, Nowlan B (2020). Control of Metastases via Myeloid CD39 and NK Cell Effector Function. Cancer Immunol Res.

[38] Bao X, Xie L (2022). Targeting purinergic pathway to enhance radiotherapy-induced immunogenic cancer cell death. J Exp Clin Cancer Res.

[39] Di Virgilio F, Vultaggio-Poma V, Sarti AC (2021). P2X receptors in cancer growth and progression. Biochem Pharmacol.

[40] Allard B, Longhi MS, Robson SC, Stagg J (2017). The ectonucleotidases CD39 and CD73: Novel checkpoint inhibitor targets. Immunol Rev.

[41] Xia C, Yin S, To KKW, Fu L (2023). CD39/CD73/A2AR pathway and cancer immunotherapy. Mol Cancer.

[42] Zhang H, Vijayan D, Li XY, Robson SC, Geetha N, Teng MWL (2019). The role of NK cells and CD39 in the immunological control of tumor metastases. Oncoimmunology.

[43] Moesta AK, Li X-Y, Smyth MJ (2020). Targeting CD39 in cancer. Nature Reviews Immunology.

[44] Thompson CB, Vousden KH, Johnson RS, Koppenol WH, Sies H, Lu Z (2023). A century of the Warburg effect. Nat Metab.

[45] Greene J, Segaran A, Lord S (2022). Targeting OXPHOS and the electron transport chain in cancer; Molecular and therapeutic implications. Semin Cancer Biol.

[46] Heiden MGV, Cantley LC, Thompson CB (2009). Understanding the Warburg Effect: The Metabolic Requirements of Cell Proliferation. Science.

[47] DeBerardinis RJ, Lum JJ, Hatzivassiliou G, Thompson CB (2008). The biology of cancer: Metabolic reprogramming fuels cell growth and proliferation. Cell Metab.

[48] Gatenby RA, Gillies RJ (2004). Why do cancers have high aerobic glycolysis?. Nature Reviews Cancer.

[49] Qiao Q, Hu S, Wang X (2024). The regulatory roles and clinical significance of glycolysis in tumor. Cancer Commun (Lond).

[50] Ancey PB, Contat C, Meylan E (2018). Glucose transporters in cancer - from tumor cells to the tumor microenvironment. FEBS J.

[51] Duan Y, Wang J, Wang J, Yang Q, Zhang Q, Lu S-Y (2022). Dual-enzyme catalytic nanosystem-mediated ATP depletion strategy for tumor elimination via excessive autophagy pathway. Chemical Engineering Journal.

[52] Li H, Li Y, Su L, Zheng K, Zhang Y, Li J (2024). Enzyme-Empowered "Two Birds with One Stone" Strategy for Amplifying Tumor Apoptosis and Metabolic Clearance. Adv Sci (Weinh).

[53] Eguchi Y, Shimizu S, Tsujimoto Y (1997). Intracellular ATP levels determine cell death fate by apoptosis or necrosis. Cancer Res.

[54] Sandulache VC, Ow TJ, Pickering CR, Frederick MJ, Zhou G, Fokt I (2011). Glucose, not glutamine, is the dominant energy source required for proliferation and survival of head and neck squamous carcinoma cells. Cancer.

[55] Navale AM, Paranjape AN (2016). Glucose transporters: physiological and pathological roles. Biophys Rev.

[56] Thorens B, Mueckler M (2010). Glucose transporters in the 21st Century. Am J Physiol Endocrinol Metab.

[57] Zambrano A, Molt M, Uribe E, Salas M (2019). Glut 1 in Cancer Cells and the Inhibitory Action of Resveratrol as A Potential Therapeutic Strategy. Int J Mol Sci.

[58] Adekola K, Rosen ST, Shanmugam M (2012). Glucose transporters in cancer metabolism. Curr Opin Oncol.

[59] Mueckler M, Kruse M, Strube M, Riggs AC, Chiu KC, Permutt MA (1994). A Mutation in the Glut2 Glucose-Transporter Gene of a Diabetic Patient Abolishes Transport Activity. J Biol Chem.

[60] Gottfried E, Lang SA, Renner K, Bosserhoff A, Kreutz M (2013). New Aspects of an Old Drug - Diclofenac Targets MYC and Glucose Metabolism in Tumor Cells. PLoS ONE.

[61] Chen WH, Luo GF, Lei Q, Hong S, Qiu WX, Liu LH (2017). Overcoming the Heat Endurance of Tumor Cells by Interfering with the Anaerobic Glycolysis Metabolism for Improved Photothermal Therapy. ACS Nano.

[62] Schormann N, Hayden KL, Lee P, Banerjee S, Chattopadhyay D (2019). An overview of structure, function, and regulation of pyruvate kinases. Protein Sci.

[63] Liang JM, Li RX, He YW, Ling CL, Wang Q, Huang YZ (2018). A novel tumor-targeting treatment strategy uses energy restriction via co-delivery of albendazole and nanosilver. Nano Res.

[64] Gallmeier WM, Osieka R, Seeber S, Bruntsch U, Hossfeld DK, Bohlandt D (1976). [Recent development in the chemotherapy of metastasizing teratocarcinomas of the testis]. Verh Dtsch Ges Inn Med.

[65] Gottschalk S, Anderson N, Hainz C, Eckhardt SG, Serkova NJ (2004). Imatinib (STI571)-mediated changes in glucose metabolism in human leukemia BCR-ABL-positive cells. Clin Cancer Res.

[66] Wang JX, Choi SYC, Niu X, Kang N, Xue H, Killam J (2020). Lactic Acid and an Acidic Tumor Microenvironment suppress Anticancer Immunity. Int J Mol Sci.

[67] Gao F, Tang Y, Liu W-L, Zou M-Z, Huang C, Liu C-J (2019). Intra/Extracellular Lactic Acid Exhaustion for Synergistic Metabolic Therapy and Immunotherapy of Tumors. Advanced Materials.

[68] Yang C, Xing S, Wei X, Lu J, Zhao G, Ma X (2024). 12-O-deacetyl-phomoxanthone A inhibits ovarian tumor growth and metastasis by downregulating PDK4. Biomed Pharmacother.

[69] Hu JJ, Liu MD, Gao F, Chen Y, Peng SY, Li ZH (2019). Photo-controlled liquid metal nanoparticle-enzyme for starvation/photothermal therapy of tumor by win-win cooperation. Biomaterials.

[70] Wang M, Wang D, Chen Q, Li C, Li Z, Lin J (2019). Recent Advances in Glucose-Oxidase-Based Nanocomposites for Tumor Therapy. Small.

[71] Fu LH, Qi C, Lin J, Huang P (2018). Catalytic chemistry of glucose oxidase in cancer diagnosis and treatment. Chem Soc Rev.

[72] Lu Z, Gao J, Fang C, Zhou Y, Li X, Han G (2020). Porous Pt Nanospheres Incorporated with GOx to Enable Synergistic Oxygen-Inductive Starvation/Electrodynamic Tumor Therapy. Adv Sci (Weinh).

[73] Ming J, Zhu T, Yang W, Shi Y, Huang D, Li J (2020). Pd@Pt-GOx/HA as a Novel Enzymatic Cascade Nanoreactor for High-Efficiency Starving-Enhanced Chemodynamic Cancer Therapy. ACS Appl Mater Interfaces.

[74] Yu J, He X, Wang Z, Liu S, Hao D, Li X (2021). Combination of starvation therapy and Pt-NP based chemotherapy for synergistic cancer treatment. J Mater Chem B.

[75] Zhao Y, Liu J, He M, Dong Q, Zhang L, Xu Z (2022). Platinum-Titania Schottky Junction as Nanosonosensitizer, Glucose Scavenger, and Tumor Microenvironment-Modulator for Promoted Cancer Treatment. ACS Nano.

[76] Hill TK, Davis AL, Wheeler FB, Kelkar SS, Freund EC, Lowther WT (2016). Development of a Self-Assembled Nanoparticle Formulation of Orlistat, Nano-ORL, with Increased Cytotoxicity against Human Tumor Cell Lines. Mol Pharmaceut.

[77] Li X, Lovell JF, Yoon J, Chen X (2020). Clinical development and potential of photothermal and photodynamic therapies for cancer. Nat Rev Clin Oncol.

[78] Premji TP, Dash BS, Das S, Chen J-P (2024). Functionalized Nanomaterials for Inhibiting ATP-Dependent Heat Shock Proteins in Cancer Photothermal/Photodynamic Therapy and Combination Therapy. Nanomaterials.

[79] Liang J, Li R, He Y, Ling C, Wang Q, Huang Y (2018). A novel tumor-targeting treatment strategy uses energy restriction via co-delivery of albendazole and nanosilver. Nano Research.

[80] Xiang QQ, Qiao B, Luo YL, Cao J, Fan K, Hu XH (2021). Increased photodynamic therapy sensitization in tumors using a nitric oxide-based nanoplatform with ATP-production blocking capability. Theranostics.

[81] Pouyssegur J, Marchiq I, Parks SK, Durivault J, Zdralevic M, Vucetic M (2022). 'Warburg effect' controls tumor growth, bacterial, viral infections and immunity - Genetic deconstruction and therapeutic perspectives. Semin Cancer Biol.

[82] Shi YF, Lim SK, Liang QR, Iyer SV, Wang HY, Wang ZL (2019). Gboxin is an oxidative phosphorylation inhibitor that targets glioblastoma. Nature.

[83] Vercellino I, Sazanov LA (2022). The assembly, regulation and function of the mitochondrial respiratory chain. Nat Rev Mol Cell Biol.

[84] Brookes PS, Yoon YS, Robotham JL, Anders MW, Sheu SS (2004). Calcium, ATP, and ROS: a mitochondrial love-hate triangle. Am J Physiol-Cell Ph.

[85] Bai R, Cui J (2023). Mitochondrial immune regulation and anti-tumor immunotherapy strategies targeting mitochondria. Cancer Lett.

[86] Vasan K, Werner M, Chandel NS (2020). Mitochondrial Metabolism as a Target for Cancer Therapy. Cell Metab.

[87] Kadenbach B (2021). Complex IV - The regulatory center of mitochondrial oxidative phosphorylation. Mitochondrion.

[88] Brzezinski P, Moe A, Adelroth P (2021). Structure and Mechanism of Respiratory III-IV Supercomplexes in Bioenergetic Membranes. Chem Rev.

[89] Sarti P, Forte E, Mastronicola D, Giuffrè A, Arese M (2012). Cytochrome c oxidase and nitric oxide in action: Molecular mechanisms and pathophysiological implications. Bba-Bioenergetics.

[90] Shiva S, Brookes PS, Patel RP, Anderson PG, Darley-Usmar VM (2001). Nitric oxide partitioning into mitochondrial membranes and the control of respiration at cytochrome oxidase. P Natl Acad Sci USA.

[91] Althaher AR, Alwahsh M (2023). An overview of ATP synthase, inhibitors, and their toxicity. Heliyon.

[92] Wan S-S, Liu M-D, Cheng Q, Cheng H, Zhang X-Z (2020). A Mitochondria-Driven Metabolic Sensing Nanosystem for Oxygen Availability and Energy Blockade of Cancer. Advanced Therapeutics.

[93] Slepnev VI, Kuznetsova LE, Gubin AN, Batrakova EV, Alakhov VY, Kabanov AV (1992). Micelles of Poly(Oxyethylene)-Poly(Oxypropylene) Block Copolymer (Pluronic) as a Tool for Low-Molecular Compound Delivery into a Cell - Phosphorylation of Intracellular Proteins with Micelle Incorporated [Gamma-32p]Atp1. Biochem Int.

[94] Rapoport N, Marin AP, Timoshin AA (2000). Effect of a polymeric surfactant on electron transport in HL-60 cells. Arch Biochem Biophys.

[95] Kabanov AV, Batrakova EV, Alakhov VY (2002). Pluronic block copolymers for overcoming drug resistance in cancer. Adv Drug Deliv Rev.

[96] Dong X, Mattingly CA, Tseng MT, Cho MJ, Liu Y, Adams VR (2009). Doxorubicin and paclitaxel-loaded lipid-based nanoparticles overcome multidrug resistance by inhibiting P-glycoprotein and depleting ATP. Cancer Res.

[97] Xiang Q, Qiao B, Luo Y, Cao J, Fan K, Hu X (2021). Increased photodynamic therapy sensitization in tumors using a nitric oxide-based nanoplatform with ATP-production blocking capability. Theranostics.

[98] Cheung EC, Vousden KH (2022). The role of ROS in tumour development and progression. Nat Rev Cancer.

[99] Arfin S, Jha NK, Jha SK, Kesari KK, Ruokolainen J, Roychoudhury S (2021). Oxidative Stress in Cancer Cell Metabolism. Antioxidants.

[100] Kim J, Kim J, Bae J-S (2016). ROS homeostasis and metabolism: a critical liaison for cancer therapy. Exp Mol Med.

[101] Moloney JN, Cotter TG (2018). ROS signalling in the biology of cancer. Semin Cell Dev Biol.

[102] Zorov DB, Juhaszova M, Sollott SJ (2014). Mitochondrial reactive oxygen species (ROS) and ROS-induced ROS release. Physiol Rev.

[103] Yang B, Chen Y, Shi J (2019). Reactive Oxygen Species (ROS)-Based Nanomedicine. Chem Rev.

[104] Rahman I, Liang B, Sajid A, Ambudkar SV, Huang H-C (2025). Photodynamic priming modulates cellular ATP levels to overcome P-glycoprotein-mediated drug efflux in chemoresistant triple-negative breast cancer. Photochem Photobiol.

[105] Wang H, Gao Z, Liu X, Agarwal P, Zhao S, Conroy DW (2018). Targeted production of reactive oxygen species in mitochondria to overcome cancer drug resistance. Nat Commun.

[106] Tang Z, Zhao P, Wang H, Liu Y, Bu W (2021). Biomedicine Meets Fenton Chemistry. Chem Rev.

[107] Li S-L, Jiang P, Jiang F-L, Liu Y (2021). Recent Advances in Nanomaterial-Based Nanoplatforms for Chemodynamic Cancer Therapy. Advanced Functional Materials.

[108] Dong Z, Hao Y, Li Q, Yang Z, Zhu Y, Liu Z (2020). Metal-polyphenol-network coated CaCO3 as pH-responsive nanocarriers to enable effective intratumoral penetration and reversal of multidrug resistance for augmented cancer treatments. Nano Research.

[109] Lin L-S, Song J, Song L, Ke K, Liu Y, Zhou Z (2018). Simultaneous Fenton-like Ion Delivery and Glutathione Depletion by MnO2-Based Nanoagent to Enhance Chemodynamic Therapy. Angewandte Chemie International Edition.

[110] Xiao J, Zhang G, Xu R, Chen H, Wang H, Tian G (2019). A pH-responsive platform combining chemodynamic therapy with limotherapy for simultaneous bioimaging and synergistic cancer therapy. Biomaterials.

[111] Yu M, Zeng W, Ouyang Y, Liang S, Yi Y, Hao H (2022). ATP-exhausted nanocomplexes for intratumoral metabolic intervention and photoimmunotherapy. Biomaterials.

[112] Ambruso SL, Gil H-W, Fox B, Park B, Altmann C, Bagchi RA (2021). Lung metabolomics after ischemic acute kidney injury reveals increased oxidative stress, altered energy production, and ATP depletion. American Journal of Physiology-Lung Cellular and Molecular Physiology.

[113] Moesta AK, Li XY, Smyth MJ (2020). Targeting CD39 in cancer. Nat Rev Immunol.

[114] Díaz-Saldívar P, Huidobro-Toro JP (2019). ATP-loaded biomimetic nanoparticles as controlled release system for extracellular drugs in cancer applications. Int J Nanomed.

[115] Ghalamfarsa G, Kazemi MH, Mohseni SR, Masjedi A, Hojjat-Farsangi M, Azizi G (2019). CD73 as a potential opportunity for cancer immunotherapy. Expert Opin Ther Tar.

[116] Vijayan D, Young A, Teng MWL, Smyth MJ (2017). Targeting immunosuppressive adenosine in cancer. Nat Rev Cancer.

[117] Feiz MS, Meshkini A (2019). Targeted delivery of adenosine 5′-triphosphate using chitosan-coated mesoporous hydroxyapatite: A theranostic pH-sensitive nanoplatform with enhanced anti-cancer effect. Int J Biol Macromol.

[118] Yang L-L, Zhang L, Wan S-C, Wang S, Wu Z-Z, Yang Q-C (2021). Two-Photon Absorption Induced Cancer Immunotherapy Using Covalent Organic Frameworks. Advanced Functional Materials.

[119] Wang M, Wu M, Liu X, Shao S, Huang J, Liu B (2022). Pyroptosis Remodeling Tumor Microenvironment to Enhance Pancreatic Cancer Immunotherapy Driven by Membrane Anchoring Photosensitizer. Adv Sci (Weinh).

[120] Pietrocola F, Pol J, Vacchelli E, Rao S, Enot DP, Baracco EE (2016). Caloric Restriction Mimetics Enhance Anticancer Immunosurveillance. Cancer Cell.

[121] Vultaggio-Poma V, Falzoni S, Chiozzi P, Sarti AC, Adinolfi E, Giuliani AL (2022). Extracellular ATP is increased by release of ATP-loaded microparticles triggered by nutrient deprivation. Theranostics.

[122] Galluzzi L, Guilbaud E, Schmidt D, Kroemer G, Marincola FM (2024). Targeting immunogenic cell stress and death for cancer therapy. Nat Rev Drug Discov.

[123] Galluzzi L, Buqué A, Kepp O, Zitvogel L, Kroemer G (2017). Immunogenic cell death in cancer and infectious disease. Nature Reviews Immunology.

[124] Ahmed A, Tait SWG (2020). Targeting immunogenic cell death in cancer. Mol Oncol.

[125] Elliott MR, Chekeni FB, Trampont PC, Lazarowski ER, Kadl A, Walk SF (2009). Nucleotides released by apoptotic cells act as a find-me signal to promote phagocytic clearance. Nature.

[126] Zhang JL, Sun XY, Zhao XF, Yang CR, Shi MH, Zhang BZ (2022). Combining immune checkpoint blockade with ATP-based immunogenic cell death amplifier for cancer chemo-immunotherapy. Acta Pharm Sin B.

[127] Zhang J, Sun X, Liu L, Zhao X, Yang C, Li K (2022). Tumor-permeated ATP-based size-controllable immunogenic cell death amplifier remodel immunosuppressive microenvironment to boost cancer immunotherapy. Applied Materials Today.

[128] Li Z, Lai X, Fu S, Ren L, Cai H, Zhang H (2022). Immunogenic Cell Death Activates the Tumor Immune Microenvironment to Boost the Immunotherapy Efficiency. Adv Sci (Weinh).

[129] Wang Y, Wang W, Gu R, Chen J, Chen Q, Lin T (2023). In Situ Vaccination with Mitochondria-Targeting Immunogenic Death Inducer Elicits CD8(+) T Cell-Dependent Antitumor Immunity to Boost Tumor Immunotherapy. Adv Sci (Weinh).

[130] Li XY, Moesta AK, Xiao C, Nakamura K, Casey M, Zhang HY (2019). Targeting CD39 in Cancer Reveals an Extracellular ATP- and Inflammasome-Driven Tumor Immunity. Cancer Discov.

[131] Mao C, Yeh S, Fu J, Porosnicu M, Thomas A, Kucera GL (2022). Delivery of an ectonucleotidase inhibitor with ROS-responsive nanoparticles overcomes adenosine-mediated cancer immunosuppression. Sci Transl Med.

[132] Deng X-C, Liang J-L, Zhang S-M, Wang Y-Z, Lin Y-T, Meng R (2024). Interference of ATP-Adenosine Axis by Engineered Biohybrid for Amplifying Immunogenic Cell Death-Mediated Antitumor Immunotherapy. Advanced Materials.

[133] Anderson AE, Parashar K, Jin K, Clor J, Stagnaro CE, Vani U (2024). Characterization of AB598, a CD39 Enzymatic Inhibitory Antibody for the Treatment of Solid Tumors. Mol Cancer Ther.

[134] Wu X, Zhang J, Deng Z, Sun X, Zhang Y, Zhang C (2025). Bacteria-based biohybrids for remodeling adenosine-mediated immunosuppression to boost radiotherapy-triggered antitumor immune response. Biomaterials.

[135] Synnestvedt K, Furuta GT, Comerford KM, Louis N, Karhausen J, Eltzschig HK (2002). Ecto-5'-nucleotidase (CD73) regulation by hypoxia-inducible factor-1 mediates permeability changes in intestinal epithelia. J Clin Invest.

[136] Giatromanolaki A, Kouroupi M, Pouliliou S, Mitrakas A, Hasan F, Pappa A (2020). Ectonucleotidase CD73 and CD39 expression in non-small cell lung cancer relates to hypoxia and immunosuppressive pathways. Life Sci.

[137] Liang L, Yang L-L, Wang W, Ji C, Zhang L, Jia Y (2021). Calcium Phosphate-Reinforced Metal-Organic Frameworks Regulate Adenosine-Mediated Immunosuppression. Advanced Materials.

[138] Sutendra G, Dromparis P, Kinnaird A, Stenson TH, Haromy A, Parker JM (2013). Mitochondrial activation by inhibition of PDKII suppresses HIF1a signaling and angiogenesis in cancer. Oncogene.

[139] Dai Z, Wang Q, Tang J, Qu R, Wu M, Li H (2022). A Sub-6 nm MnFe2O4-dichloroacetic acid nanocomposite modulates tumor metabolism and catabolism for reversing tumor immunosuppressive microenvironment and boosting immunotherapy. Biomaterials.

[140] Guo S, Han F, Zhu W (2022). CD39 - A bright target for cancer immunotherapy. Biomed Pharmacother.

[141] Wu D, Chen Q, Chen X, Han F, Chen Z, Wang Y (2023). The blood-brain barrier: structure, regulation, and drug delivery. Signal Transduct Target Ther.

[142] Priester MI, Ten Hagen TLM (2023). Image-guided drug delivery in nanosystem-based cancer therapies. Adv Drug Deliv Rev.

[143] Yang L, Yang Y, Chen Y, Xu Y, Peng J (2022). Cell-based drug delivery systems and their in vivo fate. Adv Drug Deliv Rev.

[144] Özalp VC, Schäfer T (2011). Aptamer-Based Switchable Nanovalves for Stimuli-Responsive Drug Delivery. Chem-Eur J.

[145] Sameiyan E, Bagheri E, Dehghani S, Ramezani M, Alibolandi M, Abnous K (2021). Aptamer-based ATP-responsive delivery systems for cancer diagnosis and treatment. Acta Biomater.

[146] Song X-R, Li S-H, Dai J, Song L, Huang G, Lin R (2017). Polyphenol-Inspired Facile Construction of Smart Assemblies for ATP- and pH-Responsive Tumor MR/Optical Imaging and Photothermal Therapy. Small.

[147] Biswas S, Kinbara K, Niwa T, Taguchi H, Ishii N, Watanabe S (2013). Biomolecular robotics for chemomechanically driven guest delivery fuelled by intracellular ATP. Nat Chem.

[148] Mimoto F, Tatsumi K, Shimizu S, Kadono S, Haraya K, Nagayasu M (2020). Exploitation of Elevated Extracellular ATP to Specifically Direct Antibody to Tumor Microenvironment. Cell Rep.

[149] Li H, Li Y, Zhang L, Wang N, Lu D, Tang D (2024). Prodrug-inspired adenosine triphosphate-activatable celastrol-Fe(III) chelate for cancer therapy. Sci Adv.

[150] Wang Z, Jiang Q, Dong C (2020). Metabolic reprogramming in triple-negative breast cancer. Cancer Biol Med.

[151] Yang L, Li S, Yu L, Leng J, Li N (2024). Targeting glycolysis: exploring a new frontier in glioblastoma therapy. Front Immunol.

[152] Liu B, Ma R, Zhao J, Zhao Y, Li L (2020). A smart DNA nanodevice for ATP-activatable bioimaging and photodynamic therapy. SCIENCE CHINA Chemistry.

[153] Mo R, Jiang T, DiSanto R, Tai W, Gu Z (2014). ATP-triggered anticancer drug delivery. Nat Commun.

[154] Ma Y, Adjemian S, Mattarollo SR, Yamazaki T, Aymeric L, Yang H (2013). Anticancer chemotherapy-induced intratumoral recruitment and differentiation of antigen-presenting cells. Immunity.

[155] Hou MJ, Wang ZQ, Chen JT, Tan ZK, Mao GJ, Fei JJ (2022). A Dual-Channel Fluorescent Nanoprobe for Sequential Detection of ATP and Peroxynitrite to Accurately Distinguish between Normal Cells and Cancer Cells. Anal Chem.

[156] Lu L, Li B, Ding S, Fan Y, Wang S, Sun C (2020). NIR-II bioluminescence for in vivo high contrast imaging and in situ ATP-mediated metastases tracing. Nature Communications.

[157] Gardani CFF, Cappellari AR, de Souza JB, da Silva BT, Engroff P, Moritz CEJ (2019). Hydrolysis of ATP, ADP, and AMP is increased in blood plasma of prostate cancer patients. Purinergic Signal.

[158] Morciano G, Sarti AC, Marchi S, Missiroli S, Falzoni S, Raffaghello L (2017). Use of luciferase probes to measure ATP in living cells and animals. Nat Protoc.

[159] Kitajima N, Takikawa K, Sekiya H, Satoh K, Asanuma D, Sakamoto H (2020). Real-time in vivo imaging of extracellular ATP in the brain with a hybrid-type fluorescent sensor. eLife.

[160] Lu M, Zhao X, Xing H, Xun Z, Zhu S, Lang L (2018). Comparison of exosome-mimicking liposomes with conventional liposomes for intracellular delivery of siRNA. International Journal of Pharmaceutics.

[161] Ratajczak K, Lukasiak A, Grel H, Dworakowska B, Jakiela S, Stobiecka M (2019). Monitoring of dynamic ATP level changes by oligomycin-modulated ATP synthase inhibition in SW480 cancer cells using fluorescent "On-Off" switching DNA aptamer. Anal Bioanal Chem.

[162] Zhang K, Wang Y, Xue J, Liang N, Wei Z (2023). Real-time monitoring ATP variation in human cancer organoids for a long term by DNA-based nanosensor. Anal Chim Acta.

[163] Wang Q, Sun Y, Li S, Zhang P, Yao Q (2020). Synthesis and modification of ZIF-8 and its application in drug delivery and tumor therapy. RSC Adv.

[164] Lv WX, Han ZW, Li YK, Huang YJ, Sun JS, Lu XQ (2021). Exosome-Coated Zeolitic Imidazolate Framework Nanoparticles for Intracellular Detection of ATP. Chinese J Chem.

[165] Wang DD, Qi GH, Zhou Y, Zhang Y, Wang B, Hu P (2020). Single-cell ATP detection and content analyses in electrostimulus-induced apoptosis using functionalized glass nanopipettes. Chem Commun.

[166] Nante N, Ceriale E, Messina G, Lenzi D, Manzi P (2017). Effectiveness of ATP bioluminescence to assess hospital cleaning: a review. J Prev Med Hyg.

[167] Bakke M (2022). A Comprehensive Analysis of ATP Tests: Practical Use and Recent Progress in the Total Adenylate Test for the Effective Monitoring of Hygiene. J Food Prot.

[168] Matsui A, Niimi H, Uchiho Y, Kawabe S, Noda H, Kitajima I (2019). A Rapid ATP Bioluminescence-based Test for Detecting Levofloxacin Resistance Starting from Positive Blood Culture Bottles. Sci Rep.

[169] Jacques SL (2013). Optical properties of biological tissues: a review. Phys Med Biol.

[170] Balendiran GK, Dabur R, Fraser D (2004). The role of glutathione in cancer. Cell Biochem Funct.

[171] Chai X, Fan ZT, Yu MM, Zhao J, Li LL (2021). A Redox-Activatable DNA Nanodevice for Spatially-Selective, AND-Gated Imaging of ATP and Glutathione in Mitochondria. Nano Lett.

[172] Ren H, Long Z, Cui MC, Shao K, Zhou KX, Ouyang J (2016). Dual-Functional Nanoparticles for In Situ Sequential Detection and Imaging of ATP and HO. Small.

[173] Shen YZ, Wu TT, Tian Q, Mao Y, Hu JJ, Luo XL (2019). Engineering of ATP-Powered Photosensitizer for Targeted Recycling Activatable Imaging of MicroRNA and Controllable Cascade Amplification Photodynamic Therapy. Anal Chem.

[174] Rajendran M, Dane E, Conley J, Tantama M (2016). Imaging Adenosine Triphosphate (ATP). Biol Bull.

[175] Yang DS, Ghaffari R, Rogers JA (2023). Sweat as a diagnostic biofluid. Science.

[176] Wu J, Hu S, Zhang L, Xin J, Sun C, Wang L (2020). Tumor circulome in the liquid biopsies for cancer diagnosis and prognosis. Theranostics.

[177] Lone SN, Nisar S, Masoodi T, Singh M, Rizwan A, Hashem S (2022). Liquid biopsy: a step closer to transform diagnosis, prognosis and future of cancer treatments. Mol Cancer.

[178] Nikanjam M, Kato S, Kurzrock R (2022). Liquid biopsy: current technology and clinical applications. J Hematol Oncol.

[179] Shi M, Tian Y, He L, Zhang J, Yang X, Liu H (2022). Potential roles of serum ATPase and AMPase in predicting diagnosis of colorectal cancer patients. Bioengineered.

[180] Puchades C, Sandate CR, Lander GC (2020). The molecular principles governing the activity and functional diversity of AAA+ proteins. Nat Rev Mol Cell Biol.

[181] Mao Y, Fan T, Gysbers R, Tan Y, Liu F, Lin S (2017). A simple and sensitive aptasensor for colorimetric detection of adenosine triphosphate based on unmodified gold nanoparticles. Talanta.

[182] Manjubaashini N, Sephra PJ, Nehru K, Sivakumar M, Thangadurai TD (2019). Electrochemical determination of ATP at rhodamine6G capped gold nanoparticles modified carbon felt electrode at pH 7.2. Sensor Actuat B-Chem.

[183] Huang YF, Chang HT (2007). Analysis of adenosine triphosphate and glutathione through gold nanoparticles assisted laser desorption/ionization mass spectrometry. Anal Chem.

[184] Fathi F, Rashidi MR, Omidi Y (2019). Ultra-sensitive detection by metal nanoparticles-mediated enhanced SPR biosensors. Talanta.

[185] Singh P (2016). SPR Biosensors: Historical Perspectives and Current Challenges. Sensor Actuat B-Chem.

[186] Zhao RH, Yin N, Ma LX, Zhang J, Luo YK, Guo ZM (2022). Surface Plasmon Resonance (SPR) Determination of Adenosine Triphosphate (ATP) Using Silver (I) induced Configuration Changes of a Single Stranded DNA Probe with Cytosine (C). Anal Lett.

[187] Xu Y, Li P, Zhu Y, Tang Y, Chen H, Zhu X (2022). A fluorescence nanoplatform for the determination of hydrogen peroxide and adenosine triphosphate via tuning of the peroxidase-like activity of CuO nanoparticle decorated UiO-66. Mikrochim Acta.

[188] Chu B, Wang A, Cheng L, Chen R, Shi H, Song B (2021). Ex vivo and in vivo fluorescence detection and imaging of adenosine triphosphate. J Nanobiotechnology.

[189] Li H, Kou B, Yuan Y, Chai Y, Yuan R (2022). Porous Fe3O4@COF-Immobilized gold nanoparticles with excellent catalytic performance for sensitive electrochemical detection of ATP. Biosens Bioelectron.

[190] Sianglam P, Ngamdee K, Ngeontae W (2022). Simultaneous preconcentration and fluorescence detection of ATP by a hybrid nanocomposite of magnetic nanoparticles incorporated in mixed metal hydroxide. Anal Methods-Uk.

[191] Shen YZ, Tian Q, Sun YD, Xu JJ, Ye DJ, Chen HY (2017). ATP-Activatable Photosensitizer Enables Dual Fluorescence Imaging and Targeted Photodynamic Therapy of Tumor. Anal Chem.

[192] Chen XX, Hou MJ, Wang WX, Tan M, Tan ZK, Mao GJ (2022). ATP-responsive near-infrared fluorescent nanoparticles for synergistic chemotherapy and starvation therapy. Nanoscale.

[193] Hu Y, Liu W, Fang W, Dong Y, Zhang H, Luo Q (2024). Tumor energy metabolism: implications for therapeutic targets. Mol Biomed.

[194] Tufail M, Jiang CH, Li N (2024). Altered metabolism in cancer: insights into energy pathways and therapeutic targets. Mol Cancer.

[195] Pujalte-Martin M, Belaid A, Bost S, Kahi M, Peraldi P, Rouleau M (2024). Targeting cancer and immune cell metabolism with the complex I inhibitors metformin and IACS-010759. Mol Oncol.

[196] Martin S, Dudek-Peric AM, Garg AD, Roose H, Demirsoy S, Van Eygen S (2017). An autophagy-driven pathway of ATP secretion supports the aggressive phenotype of BRAF(V600E) inhibitor-resistant metastatic melanoma cells. Autophagy.

[197] Schmitt M, Ceteci F, Gupta J, Pesic M, Bottger TW, Nicolas AM (2022). Colon tumour cell death causes mTOR dependence by paracrine P2X4 stimulation. Nature.

[198] Zhou Z, Dong D, Yuan Y, Luo J, Liu XD, Chen LY (2024). Single cell atlas reveals multilayered metabolic heterogeneity across tumour types. EBioMedicine.

[199] Chu X, Li X, Zhang Y, Dang G, Miao Y, Xu W (2024). Integrative single-cell analysis of human colorectal cancer reveals patient stratification with distinct immune evasion mechanisms. Nat Cancer.

[200] Galeano Nino JL, Wu H, LaCourse KD, Kempchinsky AG, Baryiames A, Barber B (2022). Effect of the intratumoral microbiota on spatial and cellular heterogeneity in cancer. Nature.

[201] Chen J, Larsson L, Swarbrick A, Lundeberg J (2024). Spatial landscapes of cancers: insights and opportunities. Nat Rev Clin Oncol.

[202] Pu Y, Li L, Peng H, Liu L, Heymann D, Robert C (2023). Drug-tolerant persister cells in cancer: the cutting edges and future directions. Nat Rev Clin Oncol.

